# Self‐Stabilized Supramolecular Assemblies Constructed from PEGylated Dendritic Peptide Conjugate for Augmenting Tumor Retention and Therapy

**DOI:** 10.1002/advs.202102741

**Published:** 2021-10-07

**Authors:** Xiuli Zheng, Dayi Pan, Xiaoting Chen, Lei Wu, Miao Chen, Wenjia Wang, Hu Zhang, Qiyong Gong, Zhongwei Gu, Kui Luo

**Affiliations:** ^1^ Huaxi MR Research Center (HMRRC), Department of Radiology, National Clinical Research Center for Geriatrics, Frontiers Science Center for Disease‐Related Molecular Network, State Key Laboratory of Biotherapy, West China Hospital Sichuan University Chengdu 610041 China; ^2^ National Engineering Research Center for Biomaterials Sichuan University Chengdu 610064 China; ^3^ Animal Experimental Center of West China Hospital Core Facility of West China Hospital Sichuan University Chengdu 610041 China; ^4^ West China School of Medicine West China College of Stomatology Sichuan University Chengdu 610041 China; ^5^ Amgen Bioprocessing Centre Keck Graduate Institute Claremont CA 91711 USA; ^6^ Functional and Molecular Imaging Key Laboratory of Sichuan Province Research Unit of Psychoradiology Chinese Academy of Medical Sciences Chengdu 610041 China

**Keywords:** colloidal stability, dendritic peptides, dissipative particle dynamics simulations, polymeric conjugates, supramolecular assembly, transcriptome analysis, tumor retention and therapy

## Abstract

Supramolecular self‐assemblies of dendritic peptides with well‐organized nanostructures have great potential as multifunctional biomaterials, yet the complex self‐assembly mechanism hampers their wide exploration. Herein, a self‐stabilized supramolecular assembly (SSA) constructed from a PEGylated dendritic peptide conjugate (PEG‐dendritic peptide‐pyropheophorbide a, PDPP), for augmenting tumor retention and therapy, is reported. The supramolecular self‐assembly process of PDPP is concentration‐dependent with multiple morphologies. By tailoring the concentration of PDPP, the supramolecular self‐assembly is driven by noncovalent interactions to form a variety of SSAs (unimolecular micelles, oligomeric aggregates, and multi‐aggregates) with different sizes from nanometer to micrometer. SSAs at 100 nm with a spherical shape possess extremely high stability to prolong blood circulation about 4.8‐fold higher than pyropheophorbide a (Ppa), and enhance tumor retention about eight‐fold higher than Ppa on day 5 after injection, which leads to greatly boosting the in vivo photodynamic therapeutic efficiency. RNA‐seq demonstrates that these effects of SSAs are related to the inhibition of MET‐PI3K‐Akt pathway. Overall, the supramolecular self‐assembly mechanism for the synthetic PEGylated dendritic peptide conjugate sheds new light on the development of supramolecular assemblies for tumor therapy.

## Graphical abstract

A self‐stabilized supramolecular assembly (SSA) from the PEGylated dendritic peptide conjugate is presented, which builds on the concentration‐dependent supramolecular self‐assembly that demonstrates ultrahigh colloidal stability and enhanced tumor retention to boost its photodynamic therapeutic efficiency. This study reveals the supramolecular self‐assembly mechanism of SSAs and further expands the advanced functions of dendritic peptides in the field of cancer therapy.

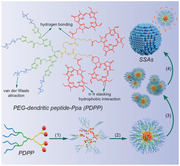

## 1 Introduction

Supramolecular peptides can be used to create a myriad of well‐organized structures with advanced functions, and they have great potential in biomedicine.^[^ ^1^ ^]^ By tailoring the sequences and external stimuli, peptide can be self‐assembled into micelles, vesicles, spheres, fibers, and tubes driven by supramolecular chemistry, and these assemblies have shown promising applications in drug delivery, gene transfection, biophotonic imaging, tissue engineering, regenerative medicine, and immunology.^[^ ^2^ ^]^ However, there are two major challenges of applying supramolecular peptides in clinical practice.^[^ ^3^ ^]^ On the one hand, unmodified peptides barely meet the requirements for fabricating controllable morphologies or performing specific functions. Covalent modifications and simple purification methods of these peptides are pursued for construction of functional supramolecular peptides.^[^ ^4^ ^]^ On the other hand, the supramolecular peptide is less

stable during the blood circulation.^[^ ^5^ ^]^ Covalent attachment or co‐assembly with polymers has been demonstrated to increase the stability of supramolecular peptides.^[^ ^6^ ^]^

To date, advances have been made in the assembly of supramolecular peptides with linear polymers.^[^ ^7^ ^]^ For example, dual self‐assembly supramolecular peptide nanotubes were built by conjugation of a carboxylic acid diblock co‐polymer with a functionalized cyclic peptide to achieve stabilization of nanotubes in water.^[^ ^5^ ^]^ Furthermore, supramolecular peptides could mimic a viral structure, and these virion‐like supramolecular assemblies could augment tumor penetration and increase the treatment efficacy.^[^ ^8^ ^]^ However, assembly of supramolecular peptides with dendrimers is still challenging.^[^ ^9^ ^]^ Notably, dendritic peptides not only possess general features of typical dendrimers but also unique properties of globular proteins.^[^ ^10^ ^]^ In our previous studies, we reported a supramolecular assembly strategy for formation bio‐inspired nano‐assemblies from dendritic peptides for highly efficient drug delivery.^[^ ^11^ ^]^ Interestingly, these highly stable supramolecular nano‐assemblies display a spherical shape in aqueous solution, and the electron density distributes evenly within each assembly unit. However, the mechanism for assembly of dendritic peptides into stable aggregates remains unveiled.

**Scheme 1:**
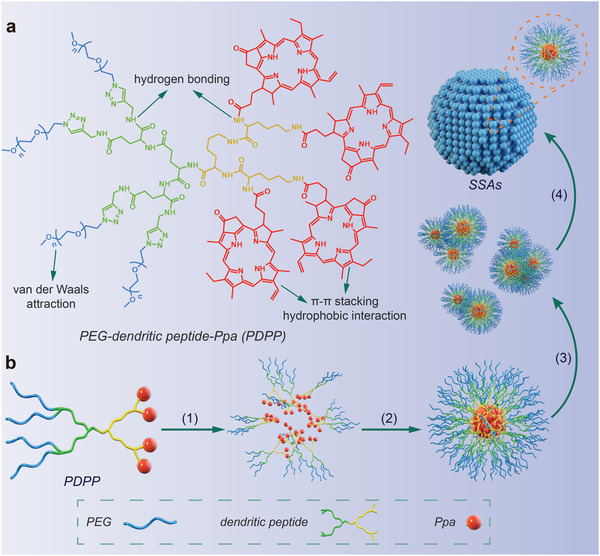
Schematic illustration for self‐assembly of self‐stabilized supramolecular assemblies (SSAs) from a PEGylated dendritic peptide conjugate, PDPP. a) Molecular structure of PDPP. b) Visualization of the supramolecular self‐assembly process of PDPP via noncovalent interactions in the aqueous phase: 1) hydrophilic PDP (PEG‐dendritic peptide) is exposed to water molecules, and hydrophobic Ppa clusters in the center to expel water molecules, 2) assembly of PDPP into unimolecular micelles as pro‐assembles, 3) gradual evolution of unimolecular micelles into oligomeric aggregates, and 4) interaction between oligomeric aggregates to form multi‐aggregates (SSAs).

Herein, we constructed self‐stabilized supramolecular assemblies (SSAs) from self‐assembly of PEGylated dendritic peptide conjugate (PEG‐dendritic peptide‐Ppa, PDPP), for exploration of the self‐assembly mechanism. To improve the stability of SSAs, the dendritic architecture is designed for SSAs to amplify noncovalent interactions of hydrophilic PEG and hydrophobic Ppa using multivalency of dendrimers. From dissipative particle dynamics (DPD) simulations, transmission electron microscopy (TEM) observations and dynamic light scattering (DLS) measurements, we proposed that PDPP could be assembled into unimolecular micelles as pro‐assembles in the aqueous solution, these unimolecular micelles may be evolved into oligomeric aggregates with an increase in the incubation time, and oligomeric aggregates could be consolidated into multi‐aggregates (**Scheme** **1**). After covalent conjugation of dendrimers, the superiorities of peptides such as biocompatibility and biosafety are maintained, which also provides a facile strategy to build SSAs with ultrahigh stability. Interestingly, spherical SSAs at 100 nm act as an excellent nanocarrier with enhancing tumor retention to stably deliver a photosensitizer (Ppa) for PDT. The results show 7.9‐fold and 3.5‐fold increases in tumor growth inhibition (TGI) in the 4T1 breast cancer and A549 non‐small cell lung cancer mouse model, respectively, after treatment with SSAs in comparison with Ppa. Transcriptome analysis demonstrated the high antitumor efficacy of SSAs is related to the inhibition of MET‐PI3K‐Akt pathway. Therefore, the PEGylated dendritic peptide conjugate could be used as a nanocontainer to deliver guest molecules (drugs, genes, or dyes) without losing high colloidal stability of nanocontainers, paving the way for wide application of these SSAs.

## 2 Results and Discussion

### 2.1 Supramolecular Self‐Assembly and Stability

**Figure 1:**
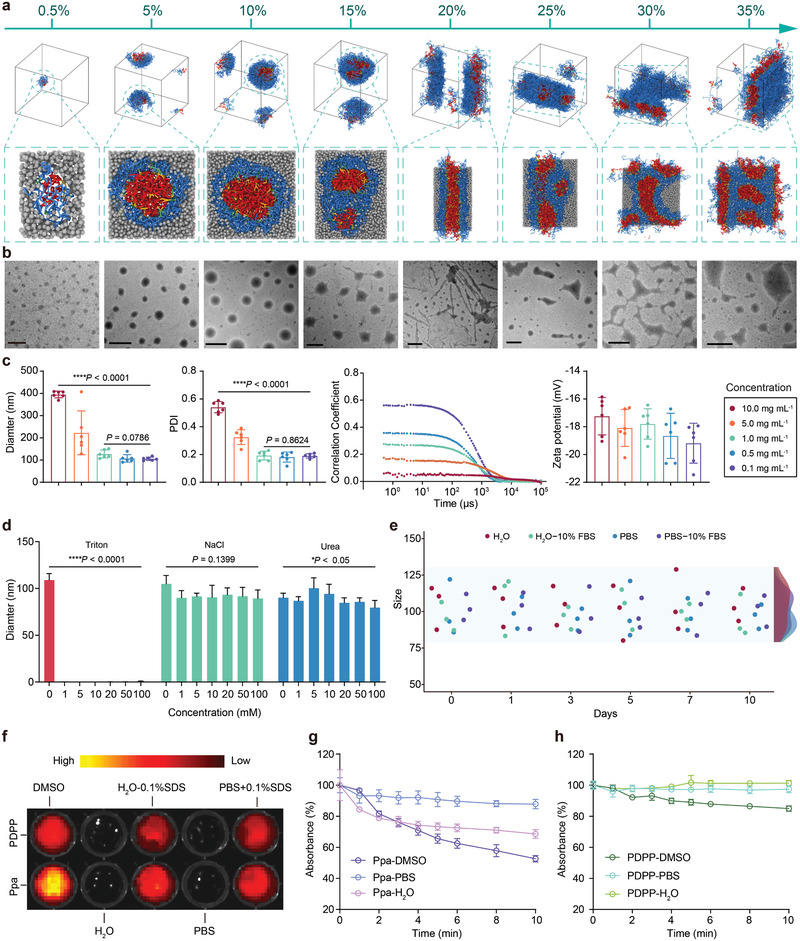
DPD simulations and physicochemical characterizations of PDPP. a) Side views and sectional views of PDPP in H_2_O at different volume fractions from 0.5% to 35% in equilibrium. Coarse‐grained blue beads for PEG, green beads for glutamic acid, yellow beads for lysine, red beads for Ppa, and gray beads for molecules of H_2_O. b) TEM images of PDPP at different concentrations. Scale bar: 0.5 µm. c) Variations of size, PDI, correlation coefficient, and zeta potential of PDPP at different concentrations (*n* = 6). d) Disassembly behavior of PDPP after dispersion in Triton, NaCl, or urea solutions (*n* = 6). e) Colloidal stability of PDPP in different solutions at 37 °C (*n* = 3). f) Fluorescence images of PDPP in different solutions. g,h) Photostability of PDPP and Ppa in different solutions after a 660 nm laser (*n* = 3, 5 mW cm^−2^).

We first used DPD simulations to study the supramolecular assembly behavior of PDPP at different volume fractions in H_2_O, as DPD simulation is currently recognized as a viable approach to intuitively study the polymeric supramolecular self‐assembly (SSA) process with evolving morphologies (Scheme S1, Supporting Information).^[^ ^12^ ^]^ With an increase in the volume fraction, PDPP undergoes morphological transition from unimolecular micelles (0.5%) to nanospheres (5–10%), cylinders (20–25%), and multigeometrical aggregates (30–35%) in sequence, and the size of these assemblies also increases (**Figure** **1a**, Figure S1 and Videos S1–S8, Supporting Information). The radial distribution function (RDF) plot shows that PDPP could encapsulate hydrophobic Ppa inside the hydrophilic PEGylated dendritic peptide conjugate at any volume fraction (Figure S2, Supporting Information). This could be attributed to the supramolecular self‐assembly process of PDPP driven by intra‐ and intermolecular noncovalent interactions, e.g., hydrophobic interaction,^[^ ^13^ ^]^ hydrogen bonding,^[^ ^14^ ^]^ and *π*–*π* stacking.^[^ ^15^ ^]^ Notably, the total potential energy of PDPP decreases as the time for the self‐assembly process prolongs or the volume fraction increases (Figure S3, Supporting Information). It implies that a lower volume fraction of PDPP could be self‐assembled into more stable assemblies. To compare the results of DPD simulations with experimental observations, PDPP was synthesized according to previously reported studies and characterized by ^1^H NMR, electrospray ionization mass spectrometry (ESI‐MS), and matrix‐assisted laser desorption/ionization time‐of‐flight mass spectrometry (MALDI‐TOF MS) (Scheme S2 and Figures S4–S13, Supporting Information).^[^ ^16^ ^]^ The drug‐loading content for Ppa in PDPP was measured via a UV–vis spectrophotometer to be 16.0 wt% (Figure S14, Supporting Information). Compared with the ^1^H NMR spectra of PDPP in DMSO‐*d*_6_, the proton signals of Ppa completely disappear in D_2_O (Figure S15, Supporting Information), revealing formation of a supramolecular self‐assembly structure from PDPP to completely hide Ppa inside PDPP. Consistent with the results of DPD simulations, transmission electron microscopy (TEM) images show that with an increase in the PDPP concentration, multifarious morphologies from nanometer scales to micrometer scales like uniform micelles, nanospheres, cylinders, and multigeometrical aggregates are generated in sequence (Figure 1b). The size distribution and zeta potential of PDPP in different solutions were subsequently determined by DLS, showing that the size and polydispersity index (PDI) increase with an increase in the PDPP concentration, while the correlation coefficient decreases as the concentration increases, but the zeta potential is not affected by the concentration, which indicates that PDPP can be self‐assembled into uniform supramolecular nano‐assemblies in the concentration range of 0.1–1.0 mg mL^−1^ (Figure 1c). All above results suggest that a concentration of 0.5 mg mL^−1^ could be used to study the supramolecular self‐assembly mechanism and stability of PDPP.

To further confirm the self‐assembly mechanism of PDPP, we investigated whether noncovalent interactions such as hydrophobic interaction, hydrogen bonding, or ionic interaction could affect supramolecular self‐assembly of PDPP.^[^ ^17^ ^]^ As observed in Figure 1d and Figure S16 (Supporting Information), no significant change in the size and PDI of PDPP after dispersion in either NaCl or urea solution at different concentrations, while the disassembly behaviors of PDPP would have occurred in the Triton solution at a high concentration through hydrophobic competition, which demonstrates that hydrophobic interaction is the key driving force for supramolecular self‐assembly of PDPP. The photographic images of PDPP in different solvents allow visualization of the impact of hydrophobic interaction on the supramolecular self‐assembly behavior (Figure S17, Supporting Information). When PDPP is dispersed in the Triton solution, PDPP exhibits a color change from yellowish green to light purple with an increase in the Triton concentration. However, no visible color change of PDPP in the NaCl or urea solution is observed, which is consistent with the DLS data. Moreover, no significant difference in the size distribution, correlation coefficient, and PDI are found for PDPP in either H_2_O or PBS, and the addition of 10% FBS has no effect on these properties (Figure S18 and Table S2, Supporting Information). Importantly, the size and PDI of PDPP in H_2_O, PBS, H_2_O or PBS with 10% FBS remain unchanged for 7 d (Figure 1e and Figure S19, Supporting Information); therefore, PDPP could hold a highly stable self‐assembly structure in a physiological condition. A low value of critical assembly concentration (CAC) of PDPP in H_2_O was measured as 4.85 µg mL^−1^ (Figure S20, Supporting Information). These results demonstrate that supramolecular assemblies of PDPP have extremely high colloidal stability, a negative surface charge and a low CAC, which may be beneficial for long blood circulation, enhanced tumor retention, and better PDT.

Meanwhile, the effect of supramolecular self‐assembly on the photophysical properties of PDPP was examined. As shown in Figures S21 and S22 (Supporting Information), PDPP exhibits very similar UV–vis absorption spectra and fluorescence emission spectra as Ppa in DMSO. However, after it is dissolved in PBS, peaks in the UV–vis absorption spectra of both PDPP and Ppa are weakened, broadened, and redshifted significantly. Therefore, the fluorescence of both PDPP and Ppa is quenched in PBS. Strong hydrophobic interaction among hydrophobic Ppa that is wrapped inside PEGylated dendritic peptide as well as aggregation of Ppa collectively contribute to strong self‐quenching of fluorescence in an aqueous solution. In contrast, when the supramolecular assembly of PDPP and aggregation of Ppa are disrupted by an anionic surfactant, sodium dodecyl sulfate (SDS), fluorescence peaks in their UV–vis absorption spectra, and fluorescence emission spectra can be detectable. Figure 1f compares the fluorescence signal of PDPP and Ppa in different solutions with or without addition of SDS, in alignment with UV–vis and fluorescence spectrophotometer measurements. It is clear that the fluorescent signal of PDPP is greatly amplified upon disassembly of the supramolecular assemblies, which is expected to occur in tumor cells. More importantly, after PDPP is dispersed in DMSO, PBS, or H_2_O, PDPP displays marked enhancement in photostability in comparison with Ppa, revealing that PDPP in either an assembly or a disassembly state possesses greater resistance against photobleaching than Ppa (Figure 1g,h). To evaluate the potential applicability of PDPP as a photosensitizer, we measured the singlet oxygen quantum yield (SOQ), molar extinction coefficient (*ε*), and fluorescence quantum yield (FQ). As presented in Figure S23 and Table S3 (Supporting Information), SOQ and FQ of PDPP are similar to those of Ppa, and *ε* of PDPP is slightly lower compared to Ppa, indicating PDPP has a potential to be a photosensitizer for PDT.^[^ ^18^ ^]^ Overall, PDPP can form spherical and uniform SSAs with excellent colloidal stability and photostability through supramolecular self‐assembly at an appropriate concentration. It suggests that the following experiments for biosafety and photodynamic therapeutic efficacy of SSAs should be in this concentration range.

### 2.2 Hemolysis and Skin Photosensitization

**Figure 2:**
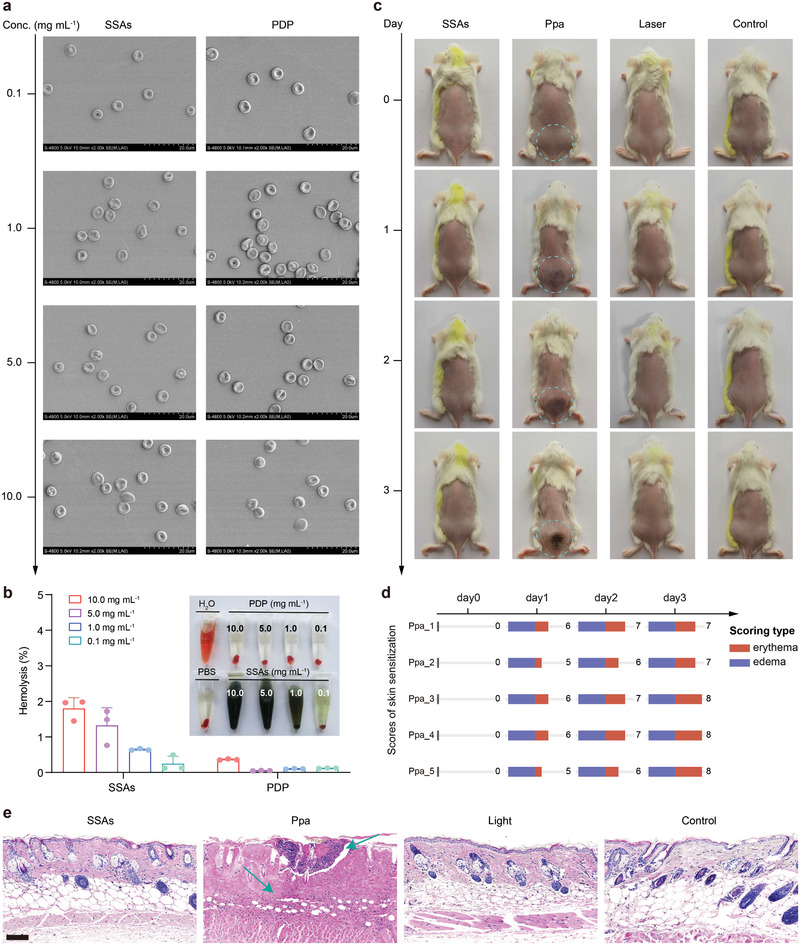
Biocompatibility and biosafety of SSAs. a) Hemocompatible effect of SSAs and PDP at different concentrations on erythrocyte aggregation and morphology via SEM. b) Hemolysis images of SSAs and PDP, and quantitative analysis results at different concentrations. c) Shaved back of BALB/c mice (*n* = 5) intravenously injected with SSAs or Ppa under 660 nm laser irradiation (10 min, 108 J cm^−2^). Circles indicate the lesion regions. d) Scores for skin response of mice receiving Ppa to determine skin photosensitization. Ppa_1–5 represent the mouse number of Ppa‐treated groups. e) H&E staining images for skin tissue harvested from mice receiving different treatments. Scale bar: 200 mm.

After evaluation of colloidal stability and photostability, we assessed biocompatibility and skin photosensitization of SSAs.^[^ ^19^ ^]^ First, mouse red blood cells (RBCs) were collected to evaluate blood compatibility of SSAs. Scanning electron microscopy (SEM) images display that SSAs and PDP (PEG‐dendritic peptide without Ppa) have no obvious impact on the biconcave disc‐like morphology of RBCs (**Figure** **2a** and Figure S24, Supporting Information). Meanwhile, no hemolysis is observed and detected even at a high concentration of SSAs and PDP (10.0 mg mL^−1^), implying that both SSAs and PDP are stealthy during blood circulation after intravenous injection of them (Figure 2b). Next, in vivo skin photosensitization of SSAs or Ppa was evaluated. ^[^ ^20^ ^]^Commonly, a 660 nm laser cannot achieve more than 10 mm depth of penetration, thus the skin structure could be impacted owing to photosensitivity. Therefore, we chose the skin of a BALB/c mouse to examine the skin photosensitivity after intravenous injection of SSAs and Ppa. The healthy mice were randomly divided into four treatment groups: i) PBS only (control), ii) laser only, iii) SSAs + laser, and iv) Ppa + laser. As shown in the images (Figure 2c,d and Figures S25–S28, Supporting Information), mice treated with Ppa exhibit rapid progression from initially slight edema into severe edema and erythema for 2 d. The edema score of the Ppa group reaches 7–8 on the third day. The histological section of skin tissues exhibits acute inflammation and necrosis with infiltration of neutrophils, congestion of small vessels, and petechial hemorrhage (Figure 2e). In contrast, mice injected with SSAs do not show any sign of skin photosensitivity. Skin tissues for PBS‐, laser‐, or SSAs ‐treated groups are also observed to have a normal morphology. The scores of skin sensitization remain zero for 3 d. Thus, the result of in vivo skin photosensitization confirms that SSAs induce a very weak skin response distinguished from Ppa, which may be due to the quenching effect of the SSAs supramolecular structure on Ppa photosensitization. Overall, these results strongly support in vivo biocompatibility and biosafety of SSAs, laying a solid foundation for its application in PDT as a photosensitizer.

### 2.3 Enhanced Cell Uptake and Tumor Retention

**Figure 3:**
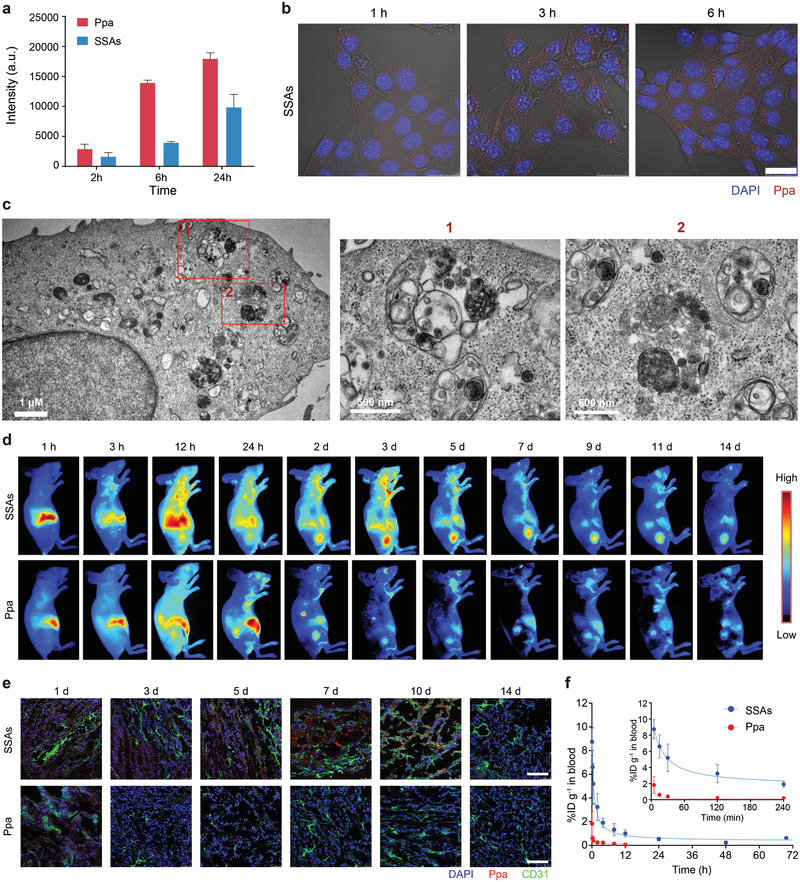
Cellular uptake, biological distribution, and pharmacokinetics of SSAs. a) Flow cytometric analysis of 4T1 cells after treatment with SSAs or Ppa for different incubation durations. b) Representative fluorescence images of 4T1 cells after incubation with SSAs. Scale bar: 25 µm. c) TEM images for the cytoplasm of 4T1 cells treated SSAs for 24 h at different scales. d) Representative in vivo fluorescence images of 4T1‐tumor‐bearing mice after intravenous injection with SSAs or Ppa. e) CLSM images of cryosections of 4T1 tumors after intravenous administration of SSAs or Ppa, including CD31 stained blood vessel channels (green), Ppa channels (red), and DAPI‐stained nucleus channels (blue). f) Elimination profiles of SSAs and Ppa in the plasma of mice receiving a single intravenous injection (*n* = 7). Scale bar: 100 µm.

It has recently been reported that nanospheres with extremely high colloidal stability can prolong blood circulation and enhance tumor retention; therefore, cellular uptake, biological distribution, and pharmacokinetics of SSAs were investigated.^[^ ^21^ ^]^ We first studied cellular uptake of SSAs in 4T1 cells by flow cytometry and confocal laser scanning microscopy (CLSM) (**Figure** **3a**,b and Figure S29, Supporting Information). Analysis of the time‐dependent endocytosis process suggests Ppa has a better uptake efficiency compared with SSAs, and cellular uptake of SSAs steadily increases over time. This is ascribed to hydrophobicity and a small molecule structure of Ppa in comparison with a big size of SSAs.^[^ ^22^ ^]^ Furthermore, compared with the control, both SSAs and Ppa are internalized and located into endosomes/lysosomes at 24 h, as exemplified in the TEM images of Figure 3c and Figure S30 (Supporting Information). These results reveal that SSAs could be effectively uptaken by 4T1 cells after a sufficient duration. Thereafter, SSAs or Ppa were injected into the tail vein of BALB/c nude mice bearing subcutaneously inoculated 4T1 breast tumors (≈50 mm^3^) to assess whether SSAs could selectively target tumor tissues. The Ppa signal was detected using a fluorescence IVIS imaging system (Figure 3d). It is observed that the fluorescence intensity of Ppa‐treated tumors decays rapidly and the fluorescence signal disappear on day 3 after injection. By contrast, the fluorescence intensity in the SSAs‐treated group remains strong on day 7 and the signal is still detectable even on day 14 after injection. Quantitative analysis of ex vivo images confirms that the Ppa from SSAs accumulated in the tumor is ≈8 times higher than that in the Ppa‐treated group on day 5 after administration (Figure S31, Supporting Information). These results are further supported from confocal images of tumor tissue sections from SSAs‐ and Ppa‐treated groups, confirming that SSAs could substantially prolong the Ppa retention time in tumors (Figure 3e). To determine the half‐life of Ppa from SSAs in the blood, we systemically administered SSAs into healthy mice and measured the drug concentration of Ppa in the plasma as a function of time after injection of SSAs (Figure 3f and Table S4, Supporting Information). A terminal half‐life of Ppa in SSAs is around 20.48 h, ≈5 times of that of Ppa (4.29 h). These results indicate that SSAs could provide a longer operation window for Ppa to enhance retention in solid tumors by prolonging the circulation time and exert an improved therapeutic effect in comparison with free Ppa.

### 2.4 In Vitro and In Vivo Antitumor Efficacy

Once we confirmed high stability and great tumor retention of SSAs, their in vitro antitumor effect to tumor cells and cytotoxicity to normal cells were evaluated. First, we evaluated the generation of ROS from SSAs and Ppa in live 4T1 cells using DCFH‐DA (DD) as a fluorescence probe for ROS.^[^ ^23^ ^]^ Both SSAs and Ppa produce a similar amount of ROS in comparison to H_2_O_2_ (Figure S32a, Supporting Information). Similar results are also obtained using flow cytometry (Figure S32b, Supporting Information). The cytotoxicity assays show consistent results (Figure S33a, Supporting Information). After irradiation by a 660 nm laser, both SSAs and Ppa display notable cytotoxicity to 4T1 cells, and a higher laser dose results in stronger cytotoxicity. We also compared the cell viability after incubation with SSAs and Ppa without light. It can be seen that Ppa without irradiation is also very cytotoxic to 4T1 cells when its concentration is higher than 1 µg mL^−1^. It is worth noting that there is no significant cytotoxicity to 4T1 cells and L02 cells treated with SSAs or PDP (Figure S33b,c, Supporting Information). There results indicate that PDP possesses great biocompatibility, and use of PDP to deliver Ppa significantly reduces the cytotoxicity of Ppa. It is expected that SSAs could effectively reduce side effects of Ppa for in vivo application.

**Figure 4:**
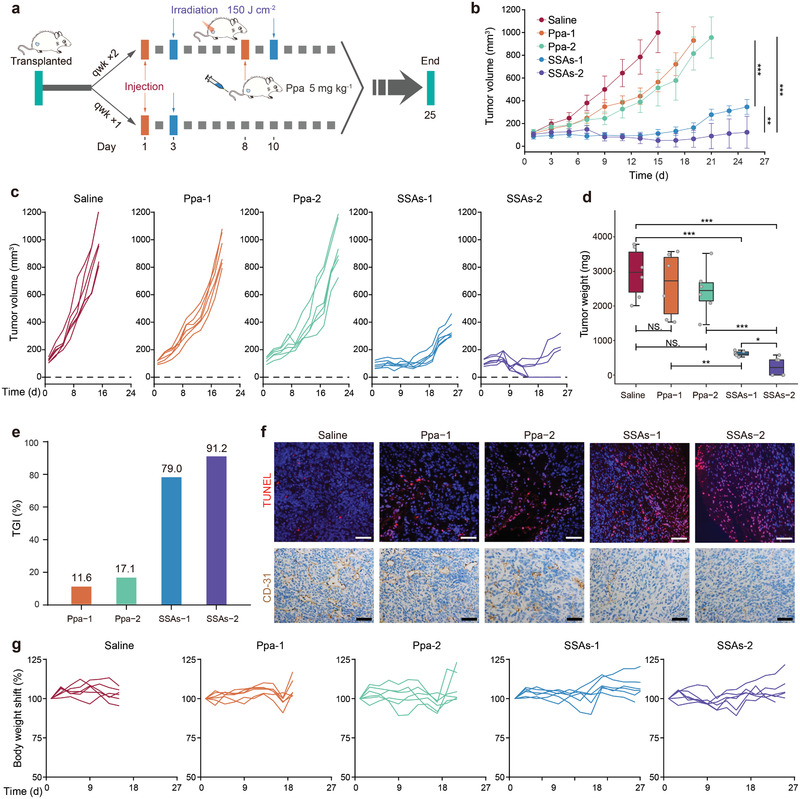
Anti‐tumor activity of SSAs in BALB/c mice bearing breast tumors. a) Schematic illustration of the in vivo treatment protocol (*n* = 6). b) Average tumor growth curves. Statistical analysis results: ***P < 0.001 for Ppa‐1 versus SSAs‐1, ***P < 0.001 for Ppa‐2 versus SSAs‐2, and **P < 0.01 for SSAs ‐1 versus SSAs‐2. c) Individual tumor growth curves. d) Tumor weights and e) TGI after different treatments. f) Images for CD31 and TUNEL staining of tumors from different groups after excision from the mice. Scale bar: 50 µm. g) Individual weight changes of mice in a subcutaneous tumor animal model over 25 d of treatments.

Encouraged by a promising in vitro photodynamic therapeutic efficacy, great biocompatibility, prolonged blood circulation as well as enhanced tumor retention, in vivo PDT of SSAs in the 4T1 mice tumor model was carried out. 4T1 cells are triple negative breast cancer cells, and they have the characteristics of strong invasiveness, high metastasis, high mortality, and poor prognosis.^[^ ^24^ ^]^ Therefore, novel and efficient treatments for breast cancer are urgently needed. To investigate the anti‐tumor efficacy and anti‐metastasis of SSAs in the 4T1 tumor model, details of once or twice treatments are shown in **Figure** **4a**. For both once and twice treatments, no significant antitumor inhibitory effect is observed in the Ppa‐treated group (Figure 4b–e), indicating that Ppa alone has a poor therapeutic effect. Notably, both once and twice treatments by SSAs substantially delay tumor growth. However, twice treatments by SSAs trigger prompt tumor regression and result in long‐term, tumor‐free survival up to at least 24 d in about 50% of the mice, a better result than other treatments. Once treatment by SSAs suppresses tumor growth, but none of these mice displays complete tumor regression. In addition, we examined the level of neovascularization and apoptosis of dissected tumor tissues after treatments (Figure 4f). Consistent with the results of Figure 4b–e, SSAs treatment can significantly increase the level of apoptosis and reduce the level of CD‐31 in tumor tissues, indicating that SSAs treatment can effectively inhibit tumor angiogenesis and promote tumor cell apoptosis. Importantly, the mice treated with SSAs behave normally, without signs of pain, stress or discomfort, and do not lose or gain weight (Figure 4g). Histological analysis of various major organs (heart, kidney, liver, lung, and spleen) from mice after treatments was conducted to detect potential biological toxicity (Figures S34 and S35, Supporting Information). No obvious pathological abnormality or inflammation is observed from both SSAs‐treated groups. In addition, much less lesions of metastasis are found in the lung and liver in the SSAs‐treat groups than those in Ppa‐treated and control groups, suggesting good prognosis of SSAs. These results support a robust antitumor efficacy against established 4T1 breast tumors and in vivo biocompatibility of SSAs, suggesting it could have great potential in its application as an antitumor therapeutic agent. Next, we assessed the therapeutic efficacy of SSAs in another A549 human non‐small cell lung cancer xenografting model to further confirm its in vitro antitumor effect (Figure S36a,b, Supporting Information). Consistent with the results of Figure 4, SSAs treatment is accompanied with negligible cytotoxicity, while Ppa has much higher cytotoxicity without laser irradiation. Treatments with both SSAs and Ppa reveal strong cytotoxicity to A549 cells under laser irradiation, and stronger toxicity is seen with an increase in either laser dose or drug concentration. Similar to experimental observations in the invasive 4T1 animal model, SSAs treatment substantially delays tumor growth in comparison with modest tumor growth suppression after Ppa treatment (Figure S36c–e, Supporting Information). Therefore, SSAs in combination with laser irradiation displays a strong antitumor effect and excellent biosafety in the treatment of highly metastatic 4T1 triple negative breast cancer and A549 non‐small‐cell lung cancer, and this SSAs‐based intervention could be translated into clinical treatment of human tumors.

### 2.5 Canonical Pathway Analysis via a Transcriptome

**Figure 5:**
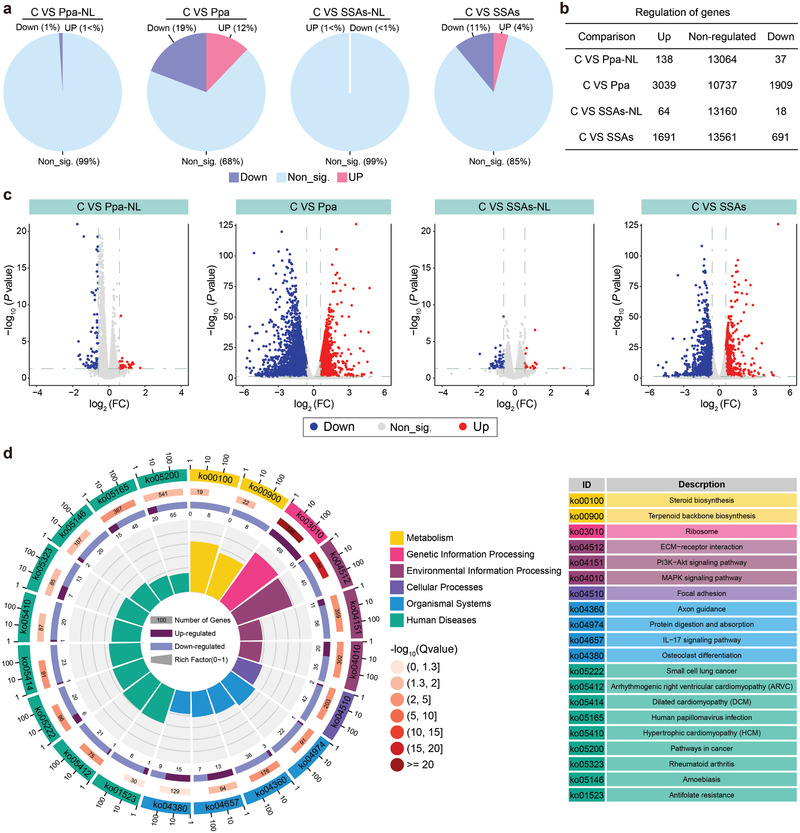
Transcriptional changes of 4T1 cells after different treatments. a) Graphic classification and b) the number of significantly or non‐significantly regulated expressed genes in 4T1 cells after different treatments compared to the control. Significance is indicated with a fold change of more than 1.5‐fold and a *P* value of <0.05 after statistical analysis (*n* = 3). Ppa‐NL: Ppa treatment without laser irradiation; SSAs‐NL: SSAs treatment without laser irradiation. c) Volcano plots of differentially expressed genes in 4T1 cells after different treatments versus the control. d) A circos image for highlighting the top 20 terms of enriched pathways after KEGG pathway enrichment analysis of significantly regulated genes in cells treated by SSAs.

**Figure 6:**
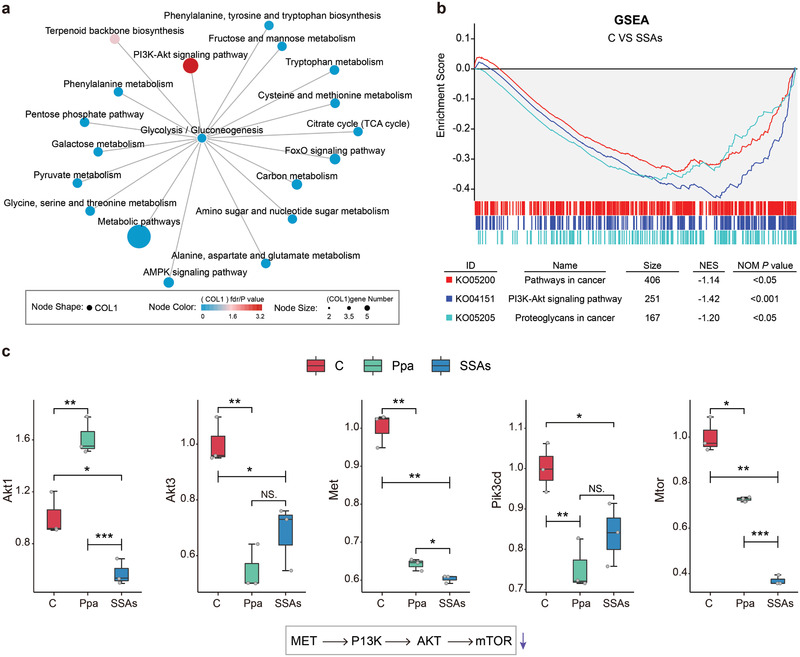
PDT effect of SSAs treatment on 4T1 cells is associated with inhibiting the MET‐PI3K ‐Akt pathway. a) Interaction networks of the enriched KEGG pathway in SSAs‐treated cells. b) Gene set enrichment analysis (GSEA) of the significantly regulated pathways in cells after SSAs treatment according to the KEGG database. The running enrichment score (ES) for the gene set is presented at the top portion. The vertical line with different colors at the bottom indicates the member of the gene set from different pathways in the ranked list of genes. Normalized enrichment score (NES) < −1 and NOM *P* value < 0.05 indicate the significantly inhibited pathways. c) Relative expression level of key genes in the MET‐PI3K‐Akt pathway through RT‐PCR analyses.

To gain a more comprehensive insight into the mechanism of therapeutic effects of SSAs at the cellular or molecular level of tumor cells, we performed the transcriptome analysis after SSAs treatments to detect the changes of gene expression at the transcriptional level.^[^ ^25^ ^]^ Without laser irradiation, very few differentially expressed genes (DEGs) are detected in cells treated with both Ppa and SSAs (**Figure** **5a**–c). However, treatment by both SSAs and Ppa with laser irradiation results in a significantly increased number of DEGs, which suggests the photodynamic therapeutic effect of SSAs could be associated with significant changes in the cellular transcriptome and laser irradiation plays an essential role in the PDT on tumor cells. Considering very few DEGs in cells treated by both Ppa and SSAs without laser irradiation (Ppa‐NL and SSAs‐NL) for the enrichment or pathway analysis, only DEGs in cells after Ppa and SSAs treatments with laser irradiation were chosen for the canonical pathway analysis to reveal SSAs‐mediated transcriptional variations (Figures S37–S42, Supporting Information). We analyzed the SSAs‐mediated DEGs according to the Kyoto Encyclopedia of Genes Genomes (KEGG) database for the enrichment analysis.^[^ ^26^ ^]^ As shown in Figure 5d and Figures S41 and S42 (Supporting Information), the results of enriched KEGG pathways indicate that most pathways are predicted to be downregulated as a higher number of downregulated enriched genes than that of upregulated genes are found in each pathway. This analysis also reveals that these DEGs are significantly enriched in cancer pathways, and these highly enriched pathways including ECM‐receptor interaction, PI3K/Akt pathway, and MAPK signaling pathway are predicted to be downregulated after treatment by SSAs with laser irradiation. Among these DEGs, more than 50 downregulated DEGs are enriched in the PI3K/Akt pathway. Examination of the interaction network of the enriched KEGG pathway indicates the downregulated PI3K/Akt pathway is the most significant one associated with metabolic pathways (**Figure** **6a**). PI3K/Akt has been identified as one of the important signaling pathways in cancer, and it controls key cellular processes of tumor cells involved in apoptosis, protein synthesis, metabolism, and cell cycle.^[^ ^27^ ^]^ Thus, the downregulating PI3K/Akt pathway may be a major cellular and signaling process in response to SSAs treatment that leads to inhibition of proliferation and survival of tumor cells. Although the enrichment pathway analyses of DEGs reveal the SSAs‐regulated process of tumor cells, key genetic variations in cells may be missing because DEGs are obtained by using a cutoff value. We used gene set enrichment analysis (GSEA) to confirm whether a priori defined set of genes in a pathway could be significantly changed (Figure 6b).^[^ ^28^ ^]^ This GSEA result also confirms that SSAs treatment results in downregulated pathways in cancer cells and PI3K‐Akt is significantly inhibited. In addition, the pathway for proteoglycans (PGs) in cancer cells is also found to be negatively regulated. Many PGs in the tumor microenvironment have been demonstrated to be key macromolecules in the tumor initiation and development stages. Some of these PGs, such as hepatocyte growth factor (HGF), are the upstream molecules to regulate the PI3K‐Akt pathway.^[^ ^29^ ^]^ *MET* gene, which expresses receptor tyrosine kinase as a receptor of HGF, is downregulated after SSAs treatment, which may result in inhibition of the upstream signal to activate the PI3K‐Akt pathway.^[^ ^30^ ^]^ The relative expression level of key genes in the Met‐PI3K‐Akt pathway is also decreased after SSAs treatment through RT‐PCR measurement, which confirms that the transcript level of the Met‐PI3K‐Akt pathway is downregulated by SSAs treatments (Figure 6c). The above results from canonical pathway analysis based on the transcriptome indicate that the photodynamic therapeutic effect of SSAs could be ascribed to the altered genes associated with tumor process, especially those for inhibiting the MET‐PI3K‐Akt pathway.

## 3 Conclusion

We have developed a PEGylated dendritic peptide conjugate PDPP to explore its supramolecular self‐assembly mechanism and then applied its self‐stabilized SSAs for cancer therapy. Increasing the concentration of PDPP during self‐assembly process results in an evolving morphology from unimolecular micelles to oligomeric aggregates and multi‐aggregates as well as an increase in the size of the self‐assembly structures. Notably, SSAs at a concentration of 0.5 mg mL^−1^ with ≈100 nm and a spherical morphology display high stability and great cyto/hemo/tissue compatibility. After treatment by SSAs with laser irradiation in both animal models with highly metastatic 4T1 triple negative breast cancer and A549 non‐small cell lung cancer, tumor retention and the photodynamic therapeutic efficacy of Ppa carried by the PEGylated dendritic peptide conjugate are significantly enhanced. Therefore, the self‐assembly mechanism provides great insight into construction of novel SSAs for improving therapeutic outcomes of photosensitizers and other therapeutic agents.

## 4 Experimental Section

Detailed experiments and methods and other relevant data are available in the Supporting Information. All animal experiments were performed in accordance with the protocol of care and use of laboratory animals, approved by the Animal Ethics Committee of West China Hospital, Sichuan University (Approval No. 2019294A). Data were presented as mean ± standard deviation (s.d.) unless otherwise indicated. The sample size was indicated in the figure caption. For data sets that were skewed distributed, the Kruskal–Wallis test was used for non‐parametric tests. For data that had a normal distribution, the Levene's test was used for homogeneity of variance (center = median), and the two‐tailed Student's *t*‐test was used for comparison between two independent samples only if the variance was equal, or the Welch's two‐sample *t*‐test was performed for adjusted analysis. Analysis of variance (ANOVA) was used for comparison of multiple samples if the variance was equal and Welch's ANOVA was used if the variance was not equal. Statistical analysis was performed using R version 4.1.0 and RStudio version 1.4.1106 with R packages multcomp, PMCMRplus, and agricolae. Significant differences were considered if *P* values < 0.05; * for *P* < 0.05, ** for *P* < 0.01, *** for *P* < 0.001, **** for *P* < 0.0001, and NS for non‐significant.

## Conflict of Interest

The authors declare no conflict of interest.

## Supporting information

Supporting Information

Supplemental Video 1

Supplemental Video 2

Supplemental Video 3

Supplemental Video 4

Supplemental Video 5

Supplemental Video 6

Supplemental Video 7

Supplemental Video 8

## Data Availability

Research data are not shared.
