# Supplementary material for: Self‐Stabilized Supramolecular Assemblies Constructed from PEGylated Dendritic Peptide Conjugate for Augmenting Tumor Retention and Therapy
Source: Adv Sci (Weinh). 2021 Oct 7;8(22):2102741. doi: 10.1002/advs.202102741 (PMC8596125; doi:10.1002/advs.202102741)
Supplement: Supplementary file 1 — Supporting Information [file ADVS-8-2102741-s007.pdf]

© 2021 Wiley-VCH GmbH

ADVANCED SCIENCE
Open Access

Supporting Information

for *Adv. Sci.*, DOI: 10.1002/advs.202102741

Self-stabilized supramolecular assemblies constructed from PEGylated dendritic peptide conjugate for augmenting tumor retention and therapy

*Xiuli Zheng, Dayi Pan, Xiaoting Chen, Lei Wu, Miao Chen, Wenjia Wang, Hu Zhang, Qiyong Gong, Zhongwei Gu, Kui Luo**

Supporting Information

Self-stabilized supramolecular assemblies constructed from PEGylated dendritic peptide conjugate for augmenting tumor retention and therapy

*Xiuli Zheng, Dayi Pan, Xiaoting Chen, Lei Wu, Miao Chen, Wenjia Wang, Hu Zhang, Qiyong Gong, Zhongwei Gu, Kui Luo**

Dr. X. L. Zheng, Dr. D. Y. Pan, Dr. W. J. Wang, Prof. H. Zhang, Prof. Q. Y. Gong, Prof. Z. W. Gu, Prof. K. Luo
Huaxi MR Research Center (HMRRC), Department of Radiology, National Clinical Research Center for Geriatrics, Frontiers Science Center for Disease-related Molecular Network, State Key Laboratory of Biotherapy, West China Hospital, Sichuan University, Chengdu, 610041, China. *E-mail: luokui@scu.edu.cn (Prof. Luo)

Prof. Q. Y. Gong, Prof. K. Luo
Functional and molecular imaging Key Laboratory of Sichuan Province, and Research Unit of Psychoradiology, Chinese Academy of Medical Sciences, Chengdu, 610041, China

X. T. Chen, L. Wu
Animal Experimental Center of West China Hospital, Core Facility of West China Hospital, Sichuan University, Chengdu, 610041, China

M. Chen
West China School of Medicine, and West China College of Stomatology, Sichuan University, Chengdu 610041, China

Prof. H. Zhang
Amgen Bioprocessing Centre, Keck Graduate Institute, Claremont, CA 91711, USA

Dr. X. L. Zheng, Prof. Z. W. Gu, Prof. K. Luo
National Engineering Research Center for Biomaterials, Sichuan University, Chengdu 610064, China

1 Experimental section

1.1 Materials and Methods

Materials

Amino acids (H-Glu(OtBu)-OtBu·HCl, Fmoc-Lys(Fmoc)-OH) and condensation agents (1-hydroxybenzotriazole (HOBt), 1-Hydroxy-7-azabenzotriazole (HOAt), O-Benzotriazole-N,N,N',N'-tetramethyl-uronium-hexafluorophosphate (HBTU), O-(7-Azabenzotriazol-1-yl)-N,N,N',N'-tetramethyluronium hexafluorophosphate (HATU)) were purchased from GL Biochem Ltd. (Shanghai, China). N, N-Diisopropylethylamine (DIPEA) and trifluoroacetic acid (TFA) were purchased from Asta Tech Pharmaceutical Co., Ltd. (Chengdu, China). Methoxy PEG Azide (mPEG-Azide, MW=2000) was purchased from Shanghai Ponsure Biotechnology Co., Ltd. Copper (II) sulfate pentahydrate ($CuSO_4·5H_2O$), sodium ascorbate and Ethylenediaminetetra-acetic acid disodium salt (EDTA-Na_2) were purchased from J&K Scientific Ltd. (Beijing, China). Pyropheophorbide-a (Ppa) from Shanghai Xianhui Pharmaceutical Co., Ltd. (Shanghai, China). Dimethyl sulfoxide-d_6 (DMSO-d_6), deuterium oxide (D_2O) and 9, 10-dimethylanthracene (DMA) were purchased from Sigma-Aldrich (Shanghai, China). 2-Propynylamine, Diethylamine, Pyrene and Sodium laurylsulfonate were purchased from Aladdin Reagents Company (Shanghai, China). Triton@X-100, Sodium Chloride (NaCl) and urea were purchased from Guangzhou saiguo biotech Co., Ltd. (Guangzhou, China). The CCK-8 kit was purchased from Dojindo Molecular Technologies, Inc., Japan.

Methods

Molecular weights were analyzed via an electrospray ionization mass spectrometry (ESI-MS, Shimadzu, Janpan) and a matrix-assisted laser desorption ionization time-of-light mass spectrometry (MALDI-TOF MS, Bruker, USA). ^{1}H-NMR spectra were recorded by a NMR spectrometer (Bruker Avance II, Germany) at 400 MHz in D_2O or DMSO-d_6. UV-Vis absorbance spectra were measured by a UV-Vis spectrophotometer (Specord 200, Analytik Jena, Germany) and fluorescence spectra by a fluorescence spectrophotometer (F-7000, Hitachi, Japan). The size and zeta potential were measured by NanoBrook Omni (Brookhaven Instruments, New York,

USA) via dynamic light scattering (DLS) methods. Morphologies of samples were observed under a transmission electron microscope (TEM, FEI Tecnai GF20S-TWIN, USA). The morphology of red blood cells (RBCs) was observed by a scanning electron microscope (SEM, S-4800, HITACHI, Japan).

1.2 Dissipative particle dynamics simulations

We constructed a coarse-grained (CG) model for dissipative particle dynamics (DPD) simulation to describe the supramolecular self-assembly process of PDPP.[1] There are five types of beads in this CG model: beads for PEG (“E”); beads for glutamic acid (“G”); beads for lysine (“L”); beads for pyropheophorbide a (“P”); and beads for solvent water molecules (“H”). The interaction parameter α_{ij} is a constant which describes the maximum repulsion between interacting beads, which are shown in Table 1. The coarse-grained model of PDPP is shown in Scheme S1, and the interaction parameters α_{ij} are presented in Table S1.

The interaction parameter α_{ij} between beads can be mapped to the Flory-Huggins χ parameter through $\Delta\alpha_{ij} = 3.27\chi_{ij}$. The parameter χ can be calculated from ***Equation (1)***:

$$\chi_{ij} = \frac{V_{ij}}{RT}(\delta_i - \delta_j)^2 \qquad \textbf{(1)}$$

Here, V_{ij} is the average molar volume of the bead, R is the gas constant, T is the absolute temperature, δ_i and δ_j are the solubility parameters for the chemical entity i and j, which can be calculated from the Materials Studio Program (Accelrys Inc.)

The location of interacting beads at each time step was obtained from Newton's equations of motion. The canonical ensemble simulations were performed in a three-dimensional box of size 30^3. The bead number density ρ was 3, and the integration time step $\Delta t = 0.02$.

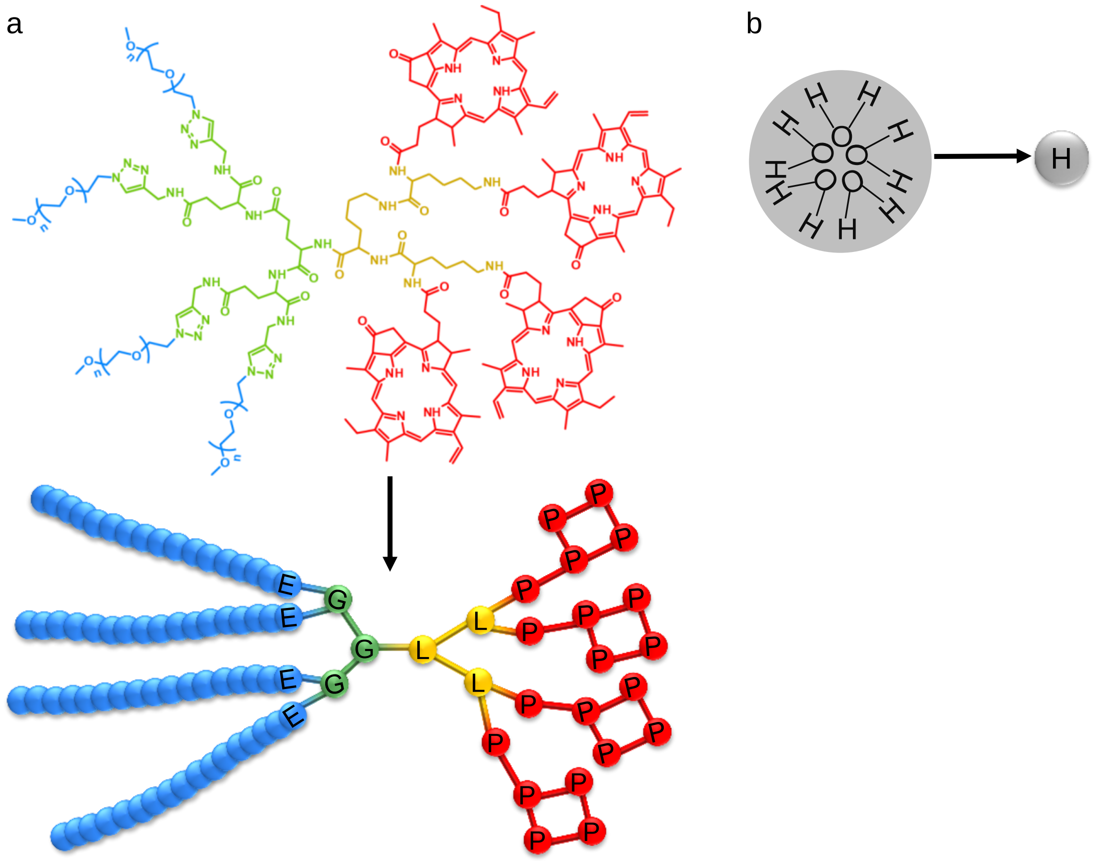

Scheme S1. (a). Molecular structure of PDPP and its corresponding coarse-grained model. (b). Molecular structure of H_2O and its corresponding coarse-grained model. The color scheme in CG model: coarse-grained seventeen blue beads E for PEG, three green beads G for glutamic acid, three yellow beads L for lysine, five red beads P for Ppa, and a grey bead H for five molecules of H_2O.

1.2 Synthesis of PEG-Peptide Dendron-Ppa (PDPP)

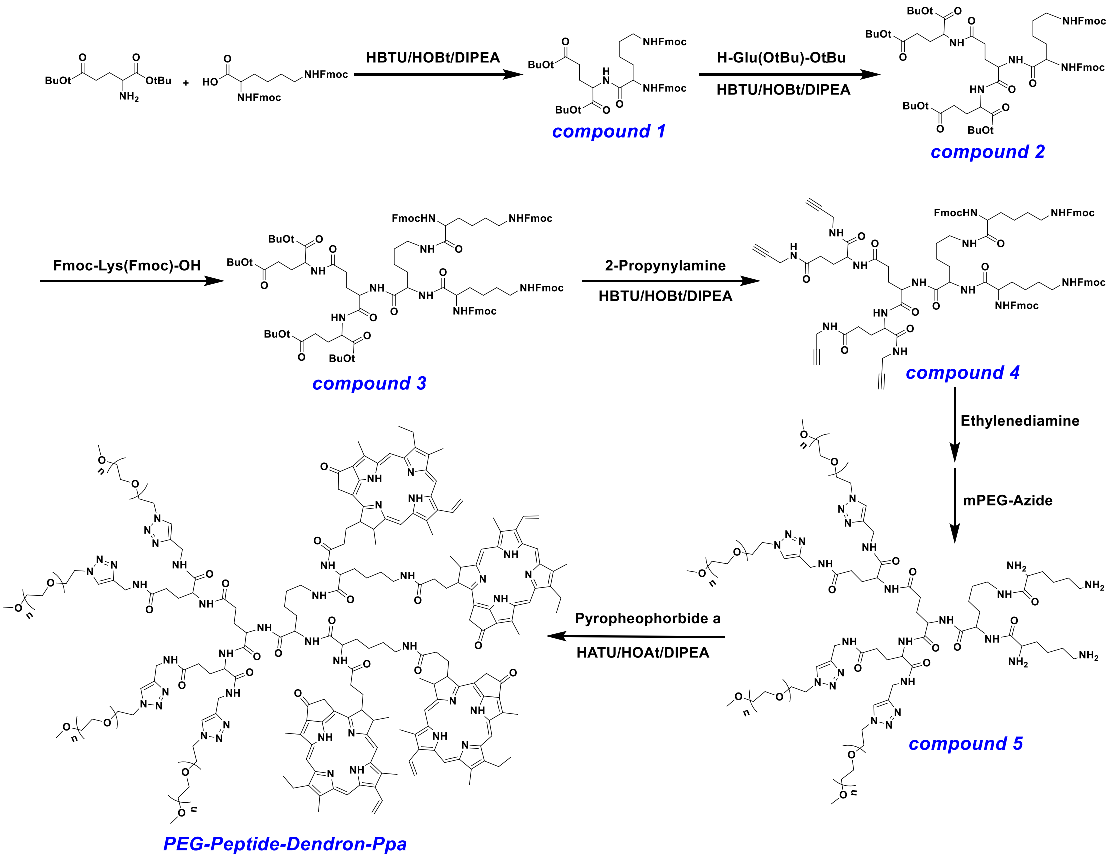

Scheme S2. Synthetic route of PEGylated dendritic peptide conjugate (PEG-dendritic peptide-pyropheophorbide a, PDPP).

Synthesis of Compound 1

Fmoc-Lys (Fmoc)-OH (4.00 g, 6.78 mmol), H-Glu (OtBu)-OtBu·HCl (3.01 g, 10.17 mmol), HOBt (1.37 g, 10.17 mmol), HBTU (3.86 g, 10.17 mmol) were added in 20 mL of anhydrous DMF, and DIPEA (3.50 g, 27.12 mmol) was added in an ice-bath. The reaction mixture was stirred at room temperature under nitrogen for 24 h. The solvent was evaporated and the residue was dissolved in EtOAc. Then the mixture was washed with saturated $NaHCO_3$, HCl (1 M) and brine. The solution was dried with $MgSO_4$, and concentrated using a rotary evaporator. The residue was subjected to column chromatography purification (silica gel, CH_2Cl_2-CH_3OH = 40: 1) to give a final yield of 74.6%.

Synthesis of Compound 2

After tert-butyl groups of ***Compound 1*** (2.16 g, 2.59 mmol) were removed by 50% TFA in DCM for 6 h, the as-prepared product was added in 30 mL of anhydrous DMF, followed by addition of H-Glu(OtBu)-OtBu·HCl (2.30 g, 7.77 mmol), HOBt (1.05 g, 7.77 mmol), HBTU (2.95 g, 7.77 mmol) and DIPEA (2.68 g, 20.72 mmol) in an ice-bath. The reaction mixture was stirred at room temperature under nitrogen for 48 h. The solvent was evaporated and the residue was dissolved in EtOAc. The mixture was washed with saturated $NaHCO_3$, HCl (1 M) and brine. The solution was dried with $MgSO_4$, and concentrated using a rotary evaporator. The residue was recrystallized in cold EtOAc to give a final yield of 80.1%.

Synthesis of Compound 3

After (Fluoren-9-ylmethoxy) carbonyl groups of ***Compound 2*** (2.08 g, 1.73 mmol) were removed by 50% DEA in DMF for 6 h, this Compound 2 without (Fluoren-9-ylmethoxy) carbonyl groups, Fmoc-Lys (Fmoc)-OH (3.06 g, 5.19 mmol), HOBt (0.70 g, 5.19 mmol), HBTU (1.97 g, 5.19 mmol) were dissolved in 40 mL of anhydrous DMF. DIPEA (1.79 g, 13.84 mmol) was added in an ice-bath. The reaction mixture was stirred at room temperature under nitrogen for 72 h. The solvent was evaporated and the residue was dissolved in EtOAc. The mixture was washed with saturated $NaHCO_3$, HCl (1 M) and brine. The solution was dried with $MgSO_4$, and concentrated using a rotary evaporator. The residue was recrystallized in cold EtOAc to give a final yield of 68.3%.

Synthesis of Compound 4

After tert-butyl groups of ***Compound 3*** (1.58 g, 0.83 mmol) were removed by 50% TFA in DCM for 6 h, Compound 3 without Tert-butyl groups, 2-Propynylamine (0.27 g, 4.98 mmol), HOBt (0.67 g, 4.98 mmol), HBTU (1.89 g, 4.98 mmol) were added in 40 mL of anhydrous DMF. DIPEA (1.72 g, 13.28 mmol) was added in an ice-bath. The reaction mixture was stirred at room temperature under nitrogen for 48 h. The solvent was evaporated and the residue was dissolved in EtOAc. The mixture was washed with saturated $NaHCO_3$, HCl (1 M) and brine. The solution was dried with

$MgSO_4$, and concentrated using a rotary evaporator. The residue was recrystallized in cold EtOAc to give a final yield of 57.8%.

Synthesis of Compound 5

After (Fluoren-9-ylmethoxy) carbonyl groups of ***Compound 4*** (0.2 g, 0.11 mmol) were removed by 50% DEA in DMF for 30 min, Compound 4 without (Fluoren-9-ylmethoxy) carbonyl groups, mPEG-Azide (1.60 g, 0.79 mmol), $CuSO_4 \cdot 5H_2O$（0.13 g, 0.53 mmol）and sodium ascorbate (0.21 g, 1.06 mmol) were added in 10 mL mixed solvent (DMSO/H_2O=4:1). The reaction mixture was stirred at 50°C under argon in the dark for 48 h. The mixture was dialyzed in the dark with 1 mM ethylenediaminetetra-acetic acid disodium salt (EDTA-Na_2) aqueous solution using a dialysis membrane (molecular weight cut-off (MWCO)=3500), and then fractionated/purified by size-exclusion chromatography using a Superose 12 HR/16/30 column on an ÄKTA FPLC system (GE Healthcare) with sodium acetate buffer containing 30% acetonitrile (pH 6.5) as a mobile phase. The mixture was dialyzed in the dark against Milli-Q water and lyophilized to give a final yield of 78.8%.

Synthesis of PEG-Peptide Dendron-Ppa (PDPP)

Compound 5 (300.0 mg, 33.2 μmol), Pyropheophorbide a (142.0 mg, 265.6 μmol), HOAt (40.7 mg, 298.8 μmol), HATU (113.6 mg, 298.8 μmol) were added in 10 mL of anhydrous DMF. DIPEA (154.4 mg, 1.20 mmol) was added in an ice-bath. The reaction mixture was stirred at room temperature under nitrogen in the dark for 48 h. The mixture was dialyzed in the dark with Milli-Q water using a dialysis membrane (MWCO = 8000), and then fractionated/purified by size-exclusion chromatography using a Superose 12 HR/16/30 column on an ÄKTA FPLC system (GE Healthcare) with sodium acetate buffer containing 30% acetonitrile (pH 6.5) as a mobile phase. The mixture was dialyzed in the dark against Milli-Q water and lyophilized to give a final yield of 52.0%.

1.3 Characterizations

1.3.1 Drug loading

Ppa was dissolved in anhydrous DMSO and then diluted in different concentrations. The absorbance values of PDPP at 667 nm were obtained and the drug loading was calculated according to the standard absorption curve. Drug loading content (DLC) = (weight of Ppa in PDPP / weight of PDPP) × 100%.

1.3.2 Size, zeta potential and morphology

The size, polydispersity index (PDI), correlation coefficient and zeta potential of self-stabilized supramolecular assemblies (PDPP) were measured by DLS, and the morphology and particle size of PDPP were observed under a TEM. Briefly, PDPP was dissolved in Milli-Q water and ultrasonically dispersed to prepare PDPP solutions at different concentrations (0.1, 0.5, 1.0, 5.0, and 10.0 mg mL^{-1}) for DLS, TEM measurements. Furthermore, the effects of hydrophobic force, ionic interaction, and hydrogen bonding on the supramolecular self-assembly of PDPP were assessed by adding Triton X-100, NaCl, and urea to the PDPP solution with the final concentrations of 1, 5, 10, 20, 50 and 100 mM for Triton X-100, NaCl, and urea. After incubation for 1 h, the size changes of PDPP were recorded by DLS.

1.3.3 Colloidal stability

The colloidal stability of PDPP was assessed by monitoring the change in the size and PDI as a function of time. PDPP were added in H_2O, PBS, H_2O containing 10% of fetal bovine serum (FBS), and PBS containing 10% of FBS respectively, and these mixtures were incubated at 37°C in a humidified chamber, and their sizes and PDI were monitored by DLS at a pre-determined time.

1.3.4 Critical assembly concentration (CAC)

The CAC of PDPP was determined by using pyrene as a fluorescence probe. Typically, 20 μL of an acetone solution of pyrene (1×10^{-4} mol L^{-1}) was transferred into a 5 mL vial. After acetone was evaporated, 2 mL of an aqueous solution of PDPP

was added into the vial to obtain the pyrene concentration of 5×10^{-5} mol L^{-1}. The excitation spectra of pyrene at different PDPP concentrations were acquired (Emission: 668 nm, Excitation: 320-360 nm, slit width: 5 nm).

1.3.5 Photophysical properties

UV-Vis absorption spectrum

In the spectroscopic studies，PDPP and Ppa were dissolved in anhydrous DMF (1 mg mL^{-1}) and then diluted 10 folds in different solutions (DMSO, DMSO+0.1%SDS, PBS, PBS+0.1%SDS). The absorption spectra of PDPP and Ppa (300-800 nm) were recorded by a UV/Vis spectrophotometer.

Fluorescence emission spectrum

To assess the self-quenching and dequenching effect of PDPP, the fluorescence emission spectra of PDPP and Ppa in different solutions (DMSO, DMSO+0.1%SDS, PBS, PBS+0.1%SDS) were obtained (Excitation: 405nm, Emission: 600-900 nm, slit width: 5 nm).

Photostability

In a 96-well plate, 100 μL aliquots of PDPP and Ppa in different solutions (DMSO, PBS and H_2O) at a Ppa concentration of 10 μg mL^{-1} were arrayed in triplicate. The absorbance at 667 nm was read on a microplate reader (ThermoFisher SCIENTIFIC). Each well was then irradiated with a 660 nm laser at 5 mW cm^{-2} at 1-min intervals for 10 min, and the absorbance value was read at pre-determined intervals. The absorbance values at 667 nm were then normalized to the pre-irradiation values and the normalized absorbance values were plotted versus the cumulative irradiation time.

Molar extinction coefficient (ε)

The molar extinction coefficient of PDPP was measured by a UV/Vis spectrophotometer. PDPP was dissolved in DMSO and then diluted to different concentrations (0.01875, 0.0375, 0.075, 0.15, 0.3, 0.6, and 1.2×10^{-6} mol L^{-1}). The absorbance values of PDPP samples at 668 nm were read and the extinction coefficient was calculated according to the Beer-Lambert law.

Fluorescent quantum yield (FQ)

PDPP and Ppa were dissolved in anhydrous toluene (6×10^{-6} mol L^{-1}). The peak area of the fluorescence emission spectrum of PDPP and Ppa was calculated (Excitation: 660 nm, Emission: 600-750 nm, slit width: 5 nm). The absorbance value of PDPP and Ppa at 600 nm was read. The fluorescent quantum yield was calculated from ***Equation (2)***:

$$\Phi_{\Delta}=\frac{P_{\mathrm{S}}}{P_{\mathrm{R}}}\times\frac{A_{\mathrm{R}}}{A_{\mathrm{S}}}\times\mathrm{ref.}\Phi_{\Delta} \quad \textbf{\textit{(2)}}$$

Where A is the absorbance at an excitation wavelength of 660 nm, and P is the peak area of the fluorescence emission spectrum. S refers to the sample and R to the reference. The FQ (Φ_{Δ}) of each sample is calculated from the reference Φ_{Δ} value, 0.30 for Ppa.

Singlet oxygen quantum yield (SOQ)

The amount of singlet oxygen generated from PDPP and Ppa was detected using 9, 10-dimethylanthracene (DMA) as a singlet oxygen sensor. PDPP and Ppa were dissolved in DMSO and added to a DMA stock solution to obtain a final concentration of 25 μM DMA. Each sample was added into a 96-well plate, followed by laser irradiation (80 mW cm^{-2}) at a wavelength of 660 nm. The fluorescence intensity of DMA (excitation at 360 nm; emission 440 nm) was measured over time. The singlet oxygen quantum yield was calculated from ***equation (3)***:

$$\Phi_{\Delta}=\frac{K_{\mathrm{S}}}{K_{\mathrm{R}}}\times\frac{A_{\mathrm{R}}}{A_{\mathrm{S}}}\times\mathrm{ref.}\Phi_{\Delta} \quad \textbf{\textit{(3)}}$$

Where A is the absorbance at 660 nm (the excitation wavelength), and K is the gradient of the DMA intensity curve. S refers to the sample and R to the reference. The SOQ (Φ_{Δ}) of each sample is calculated from the reference Φ_{Δ} value, 0.50 for Ppa.

1.3.6 Cell culture

4T1 cell line derived from the BALB/c spontaneous mammary carcinoma, human normal hepatocyte (L02 cell line) and human non-small cell lung tumor (A549 cell line) were selected for this study. All these cell lines were purchased from Chinese

Academy of Science Cell Bank for Type Culture Collection (Shanghai, China) and cultured in RPMI-1640 medium, supplemented with 10% fetal bovine serum (FBS), penicillin G (100 U mL^{-1}) and streptomycin (100 μg mL^{-1}), and incubated in 5% CO_2 atmosphere at 37°C.

1.3.7 *In vitro* ROS generation

The intracellular ROS generation was analyzed by using a peroxide-sensitive fluorescent probe, 2′, 7′ - dichlorofluorescin-diacetate (DCFH-DA, Beyotime, Beijing, China). According to the manufacturer's instruction, cells (1×10^6 cells per well) were treated SSAs and Ppa at the same concentration of 1 μg Ppa mL^{-1}. After 24 h incubation, the spent medium was discarded and fresh one added, and each well was then irradiated using a 660 nm laser with 1 J cm^{-2}. After cells were incubated with 1.0 μM DCFH-DA at 37°C for 40 min, they were harvested and suspended in 100 μL of PBS for flow cytometer analyses. 20,000 events were collected and Flowjo software was used for quantifying the intensity of fluorescence.

1.3.8 Cytotoxicity

The CCK-8 assay was used to examine the photodynamic therapeutic effect of SSAs on cell viability against three cell lines. 4×10^3 4T1 cells per well were seeded in a 96-well plate, and then incubated overnight. The cells were treated with different concentrations of Ppa and SSAs at an equivalent Ppa dose. After 24 h incubation, the medium was replaced with fresh one, and the wells were irradiated using a 660 nm laser with 1 J cm^{-2}. After 24 h, the spent media was replaced and the cell viability was assessed using the CCK-8 reagent. The absorbance at 480 nm of each well was recorded on a microplate reader. Untreated cells were served as a control. Results were presented as the average cell viability by following the manufacturer's instruction. IC50 values were calculated by using nonlinear regression in software Graphpad Prism7. The cytotoxicity of SSAs and Ppa with or without laser against A549 cell line was also measured through the same procedure.

The cytotoxicity of SSAs and PDP without laser irradiation was also measured by

CCK-8 assay. 5×10^3 L02 normal cells per well were seeded in a 96-well plate, and then treated by Ppa and SSAs for 24 h incubation or PDP for 24 h and 48 h incubation. Cell viability of each group was obtained through the same process.

1.3.9 *In vitro* cellular uptake

To determine cellular uptake and intracellular distribution of SSAs, 4T1 cells (2×10^4) were seeded on each glass coverslip to allow attachment, and incubated with Ppa and PDPP for 1 h, 3 h and 6 h, while Ppa was used as a control. After nuclear staining by Hoechst 33258, cells were washed by PBS and then observed under a confocal laser scanning microscopy (CLSM) (excitation and emission wavelengths: 630 nm and 678 nm), and images were captured by Leica Confocal Software.

For quantitative characterization of uptake of SSAs by cells, 4T1 cells were seeded in 6 well plates at 1×10^5 cells per well and incubated for 2 h, 6 h and 24 h. After the spent medium was replaced with fresh one, cells were treated by Ppa and SSAs at an equivalent concentration of 1 μg Ppa mL^{-1} for pre-specified times. Cells were then washed with PBS twice, harvested, and suspended in 100 μL of PBS for flow cytometer analyses. 20,000 events were collected and Flowjo software was used for generating each histogram.

For TEM, 4T1 cells cultured in 6 cm dishes were incubated with Ppa and SSAs. After incubation, cells were rinsed and fixed with 5% glutaraldehyde in sodium cacodylate buffer at room temperature, and then harvested for acquiring TEM images.[2]

1.3.10 Animals and the tumor model

Female BALB/c mice or nude mice (18-22 g) were purchased from Chengdu Dashuo Biological Technology (Chengdu, China). All animal experiments were performed in accordance with the protocol of the care and use of laboratory animals, approved by the Animal Ethics Committee of West China Hospital, Sichuan University (Approval No. 2019294A). Mice were inoculated with 5×10^5 4T1 cells subcutaneously on the right flank for the 4T1 model, and nude mice were inoculated

with 6×10^5 A549 cells subcutaneously on the right flank for the A549 model. About 2 weeks after implantation, mice with a tumor volume size of 100 mm^3 were used for biodistribution and imaging, and mice with a tumor volume size of 50 mm^3 for antitumor studies.

1.3.11 Blood compatibility

Erythrocytes morphologies and aggregation

Erythrocytes were collected from female BALB/c mice, and erythrocytes suspension was prepared by centrifuging the citrated whole blood at 1000 g for 5 min at room temperature. After the plasma and buffy coat layers were removed, the erythrocytes were washed with PBS 3 times. 20 μL erythrocytes were mixed with 100 μL of SSAs or PDP (PEG-dendritic peptide) dispersion in the PBS buffer for a final concentration of 0.1, 1.0, 5.0 and 10.0 mg mL^{-1} after vortexing. A PBS buffer solution with an equal volume was used as a control. The mixture was incubated for 15 min, washed with PBS and fixed with 4% paraformaldehyde overnight. The suspensions were dropped onto glass slides, dehydrated with 55, 65, 75, 85, 95, and 100% (v/v) ethanol, sequentially, for 10 min, and then dried overnight in air at room temperature. The dried erythrocytes samples were coated with gold and analyzed under a SEM.

Hemolysis of erythrocytes

Erythrocytes were diluted with PBS to prepare the erythrocyte suspension. 2 v/v % PDP and SSAs (1 mL) at three concentrations (0.1, 1.0, 5.0 and 10.0 mg mL^{-1}) was mixed with the erythrocyte suspension (50 μL) and the mixture was incubated at 37°C for 12 h. After centrifugation at 1000 g for 15 min, 200 μL of the supernatant was added into a 96-well plate. The absorbance of the supernatant was measured with a Microplate Reader at 540 nm, and hemolysis rate was determined using the following ***Equation (4)***:

$$\text{Hemolysis rate (\%)} = \frac{A_S - A_0}{A_{100} - A_0} \times 100 \quad \textbf{(4)}$$

Where A_S is the absorbance of the sample, A_{100} is the absorbance of lysed erythrocytes in H_2O (a positive control), and A_0 is the absorbance of 0% hemolysis in PBS (a negative control).

1.3.12 Skin photosensitization

The backs of healthy BALB/c mice were depilated 3 days before treatment to ensure that all injures had recovered. The shaved mice were randomly divided into 4 groups: (a) SSAs + laser irradiation; (b) Ppa + laser irradiation; (c) laser irradiation; (d) control (without any treatment). The dose of Ppa was 5 mg Ppa kg^{-1}. Right after administration, the anesthetized mice were irradiated by a 660 nm laser (0.26 W cm^{-2}, 10 min, 108 J cm^{-2}). The spleen was covered with tinfoil to avoid the expose to the laser. The skin on the back of the mice was photographed on day 0, day 1, day 2 and day 3 after irradiation. The degree of skin response was examined and scored according to Table S5, and Paraffin sections of the skin were stained with hematoxylin and eosin (H&E) according to the manufacturer's instructions.[3]

1.3.13 *In vivo* pharmacokinetics

Healthy pathogen-free BALB/c mice (5-6 weeks, female) were used to determine the pharmacokinetics of SSAs (n = 7). The mice were intravenously injected with Ppa and SSAs (5 mg Ppa kg^{-1}), respectively. Blood (20 μL) was collected at different time points (5 min, 15 min, 30 min,1 h, 2 h, 4 h, 6 h, 8 h, 12 h, 24 h, 36 h, 48 h and 72 h). Plasma was diluted to 50-fold with DMSO for fluorescent measurements using a BioTek plate reader. The pharmacokinetic parameters were calculated using a two-compartment model by WinNonlin 5.2 software.

1.3.14 *In vivo* fluorescence imaging and biodistribution

In vivo fluorescent imaging

After BALB/c nude mice developed established tumors (100 mm^3), saline, Ppa and SSAs (5 mg Ppa kg^{-1}) were intravenously injected into mice via their tail veins (n = 3). Fluorescence signals were detected at 1 h, 3 h, 12h, 24 h, 2 d, 3 d, 5 d, 7 d, 9 d, 11 d

and 14 d post-intravenous injection by a small-animal in vivo fluorescence imaging system (CRi Maestro EX, USA) with excitation at 450 nm and emission at 560 nm. The mean fluorescence intensity (MFI) was quantitated by CRi Maestro EX 3.0.

Ex vivo fluorescent imaging

After BALB/c mice developed established tumors (100 mm^3), Ppa and SSAs (5 mg Ppa kg^{-1}) were intravenously injected into mice via their tail veins (n = 3). The mice were sacrificed at different time points (1 d, 3 d, 5 d, 7 d, 10 d and 14 d) after post-injection, and tumors were excised for *ex vivo* imaging and semi-quantitative distribution analysis by an *in vivo* imaging system (IVIS Spectrum In Vivo Imaging System, USA). These tumors were excised for preparing frozen sections. The fixed tumor samples were sectioned into 5 μm-thick slides and incubated with Alex Fluor 594 labeled anti-CD31 antibody (Abcam, UK) and DAPI (Dojindo, Japan). A confocal microscope was used to observe accumulation and retention of Ppa in the collected solid tumor.

1.3.15 *In vivo* photodynamic therapy

In the 4T1 breast tumor model, when the tumor volume reached about 50 mm^3, the mice were divided into 5 groups (n = 6) and injected intravenously with saline, Ppa-1(one treatment), Ppa-2 (two treatments), SSAs-1 (one treatment) and SSAs-2 (two treatments) every four days. The dose per injection was 5 mg Ppa kg^{-1}. After 3 d post-injection, mice were anesthetized with 2% (v/v) isofluorane and tumors were irradiated for 8 min (660 nm, 200 mW cm^{-2}); The mice were monitored daily to identify any signs of toxicity, while the tumor volumetric size and the body weight were measured every 2 d. Tumor volumes were calculated using the following formula: tumor volume = length × width^2 × 0.5. Mice were euthanized at a defined end point or when the tumor diameter reached 10 mm. To evaluate laser ablation induced by laser therapy, the mice were sacrificed on day 25 after treatment, and main organs (heart, liver, spleen, lung and kidney) were harvested for Hematoxylin-Eosin (H&E) staining. The tumors harvested from all groups were photographed, weighed

and stained by the terminal deoxynucleotidyl transferase dUTP nick end labeling (TUNEL) and CD31 for histology studies.

For studies with an A549 human non-small cell lung tumor model, nude mice were randomly allocated into three groups (n= 5): saline (control), Ppa, and SSAs when the average tumor volume reached 50 mm^3. Saline, Ppa, and SSAs (5 mg Ppa kg^{-1}) were injected intravenously on day 1, 14, 21 and 28. At 3 d post injection, the tumors from groups Ppa, and SSAs were irradiated with a 660 nm laser (200 mW cm^{-2}, 8 min). The tumor growth was monitored and the tumor size was measured every 3 d. The mice were sacrificed on day 61 after treatment.

1.3.16 RNA-seq and enriched signaling pathways analysis

4T1 cells (10^6/culture) were treated by SSAs and free Ppa with or without laser. Untreated cells were set as a control. Total RNA was extracted (TRIzol Reagent, Thermo Fisher Scientific, USA) and rRNAs were removed by Ribo-zero™ rRNA Removal Kit (Epicentre, CA, USA). Relative abundance of transcripts was calculated with kallisto. The raw RNA-Seq count data were processed by DESeq R packages to obtain differentially expressed genes (DEGs).[4] RNA sequences were aligned to reference annotations for the Mus musculus genes (GRCm38.P4). A cutoff fold change of >1.5 or <−1.5 and a P value of <0.05, which were obtained by comparing each treated sample to the control, were applied for selecting significant DEGs. Enrichment analyses were performed according to the KEGG database. For gene set enrichment analysis (GSEA), all gene sets were analyzed against the curated gene sets from MSigDB to identify significantly enriched signaling pathways by using the default parameters of GSEA software. Experiments were performed in biological replicates (n=3).

1.3.17 Statistical analysis

Data were presented as mean ± s.d. unless otherwise indicated. Sample size was indicated in figure captions. For datasets that were skewed distributed, the Kruskal-Waliis test was used for non-parametric tests. For data that had a normal

distribution, the Levene's Test was used for Homogeneity of Variance (center = median), and the two-tailed Student's t-test was used for comparison between two independent samples only if the variance was equal, or the Welch's Two Sample t-test was performed for adjusted analysis. ANOVA was used for comparison of multiple samples if the variance was equal and Welch's ANOVA was used if the variance was not equal. Statistical analysis was performed using R version 4.1.0 and RStudio version 1.4.1106 with R packages multcomp, PMCMRplus and agricolae. Significant differences were considered if P values < 0.05; * for P < 0.05, ** for P < 0.01, *** for P < 0.001, **** for P < 0.0001 and NS. for non-significant.

2 Results

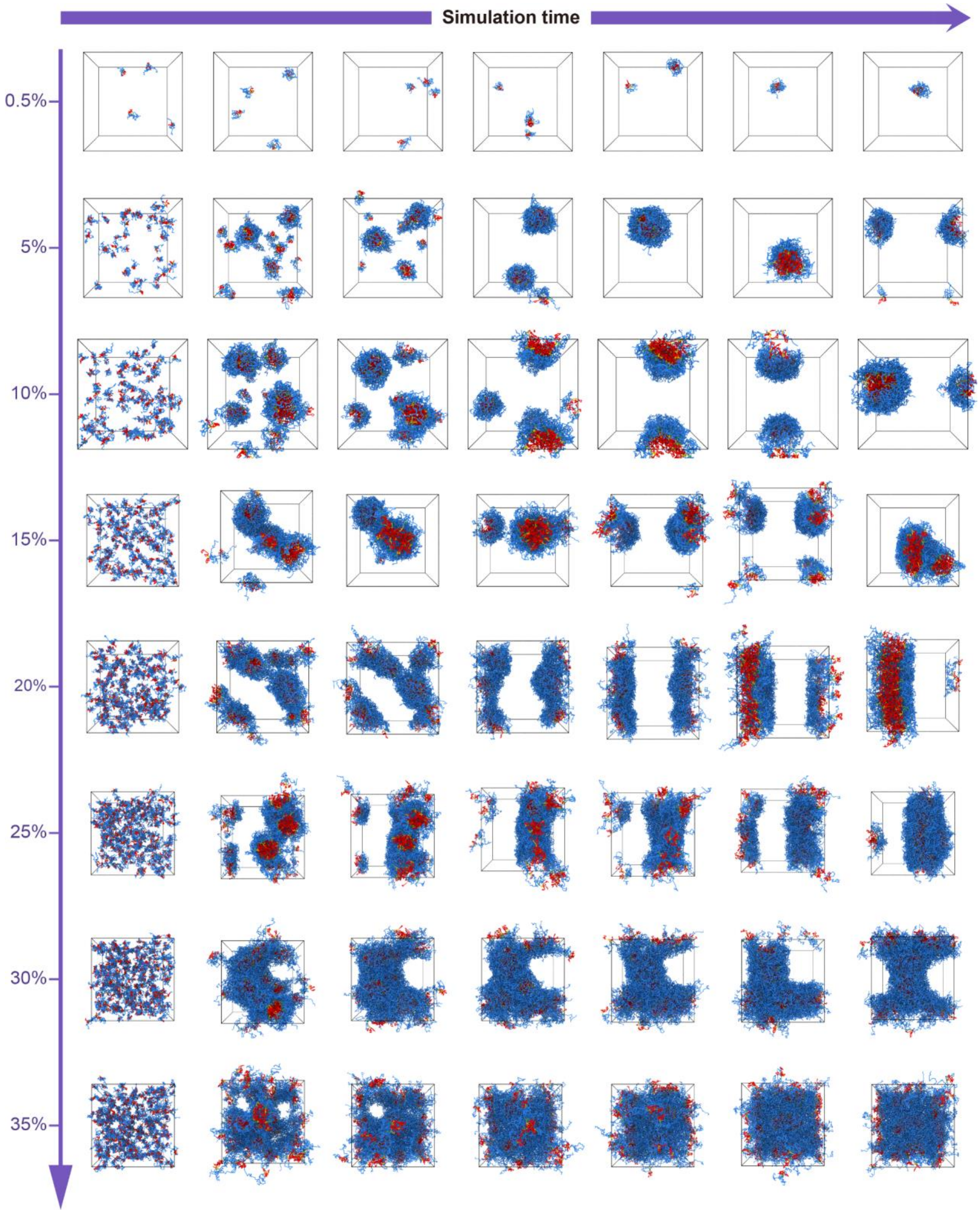

Figure S1. DPD simulations of supramolecular self-assembly process for PDPP in different volume fractions. Coarse-grained blue beads for PEG, green beads for glutamic acid, yellow beads for lysine, and red beads for Ppa.

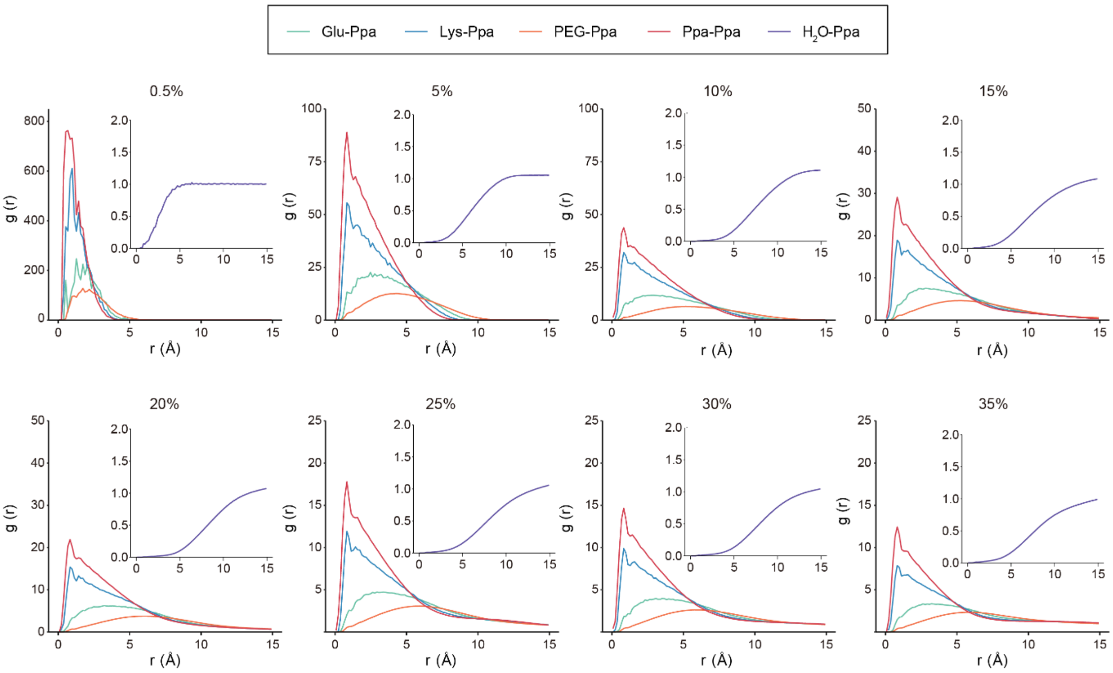

Figure S2. Radial distribution functions of interaction between Glu-Ppa, Lys-Ppa, PEG-Ppa, Ppa-Ppa, and H_2O-Ppa in PDPP at different volume fractions.

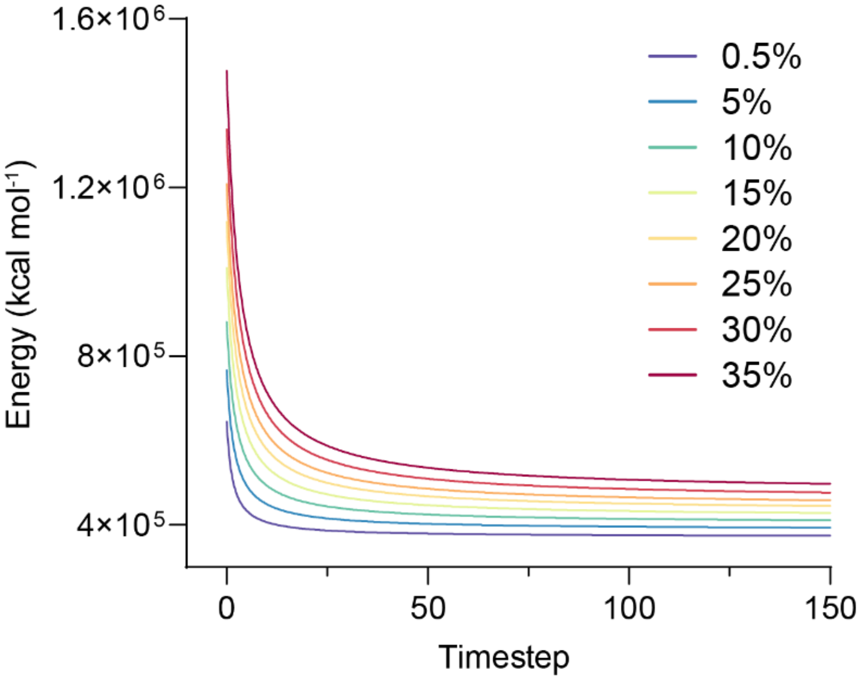

Figure S3. Temporal evolution of total potential energy of PDPP at different volume fractions. The total potential energy decreases during the self-assembly process. The result suggests that a simulation time of 50 ps is long enough for PDPP to reach a steady-state, and a lower volume fraction of PDPP could be more effective to form a more stable structure at a reduced and lower energy level.

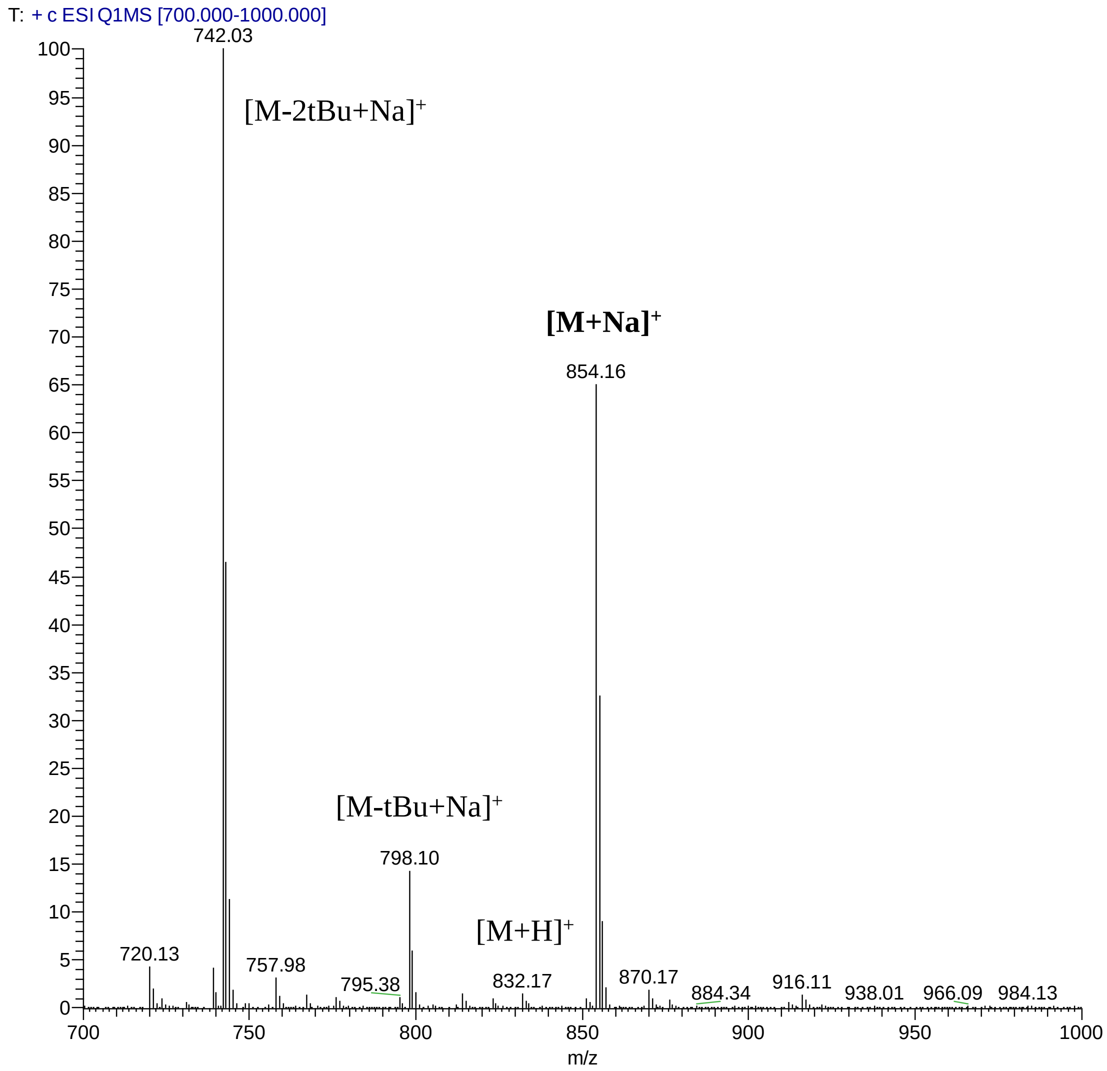

Figure S4. ESI-MS of ***Compound 1*** ([M + Na]$^{+}$): 854.41(calculated), 854.16 (found).

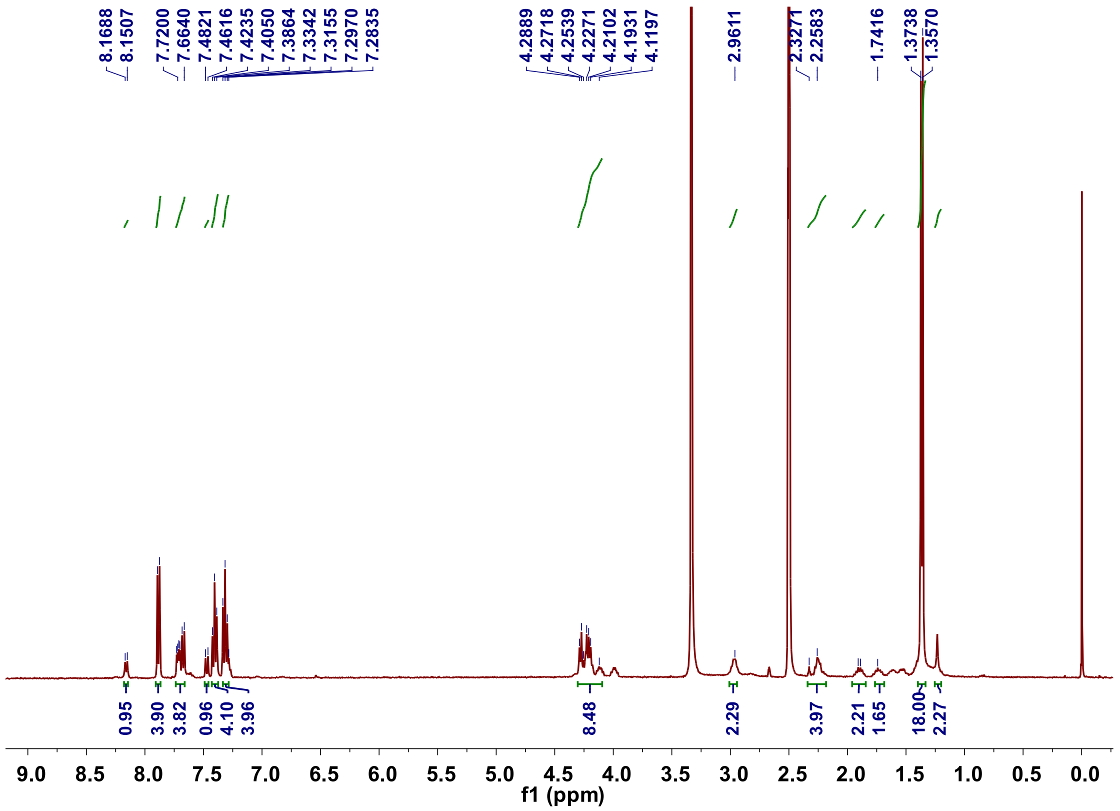

Figure S5. ^{1}H NMR spectra of ***Compound 1***. ^{1}H NMR (400 MHz, DMSO-d_6): δ 8.16 (d, J = 7.2 Hz, 1H), 7.88 (d, J = 7.5 Hz, 4H), 7.75 – 7.66 (m, 4H), 7.47 (d, J = 8.2 Hz, 1H), 7.40 (t, J = 7.4 Hz, 4H), 7.31 (m, 4H), 4.22 (m, 8H), 2.96 (s, 2H), 2.29 (d, J = 27.5 Hz, 4H), 1.90 (d, J = 7.7 Hz, 2H), 1.74 (s, 2H), 1.37 (d, J = 6.8 Hz, 18H), 1.23 (s, 2H).

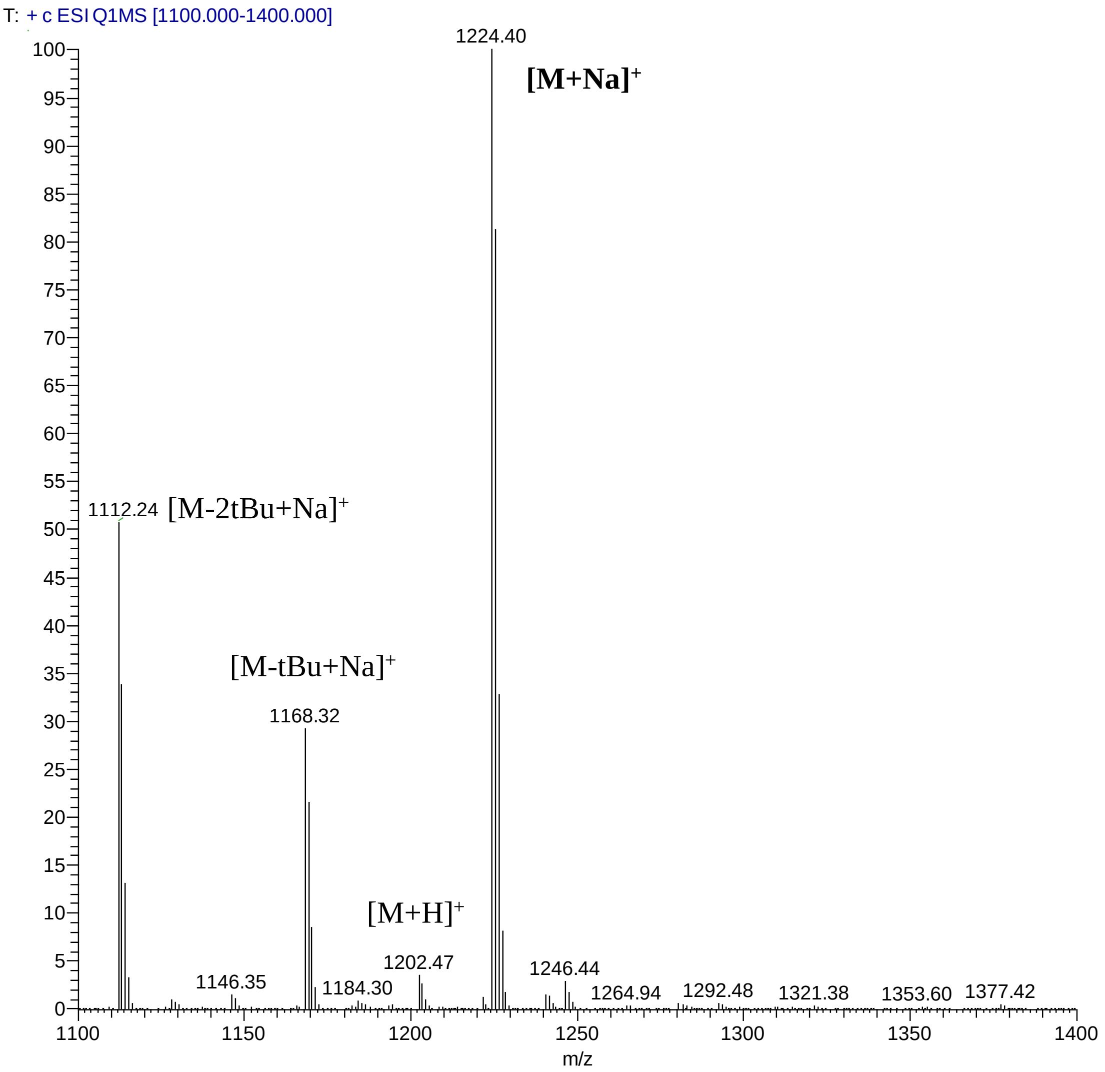

Figure S6. ESI-MS of ***Compound 2*** (m/z, $[M + Na]^+$): 1224.62 (calculated), 1224.40 (found).

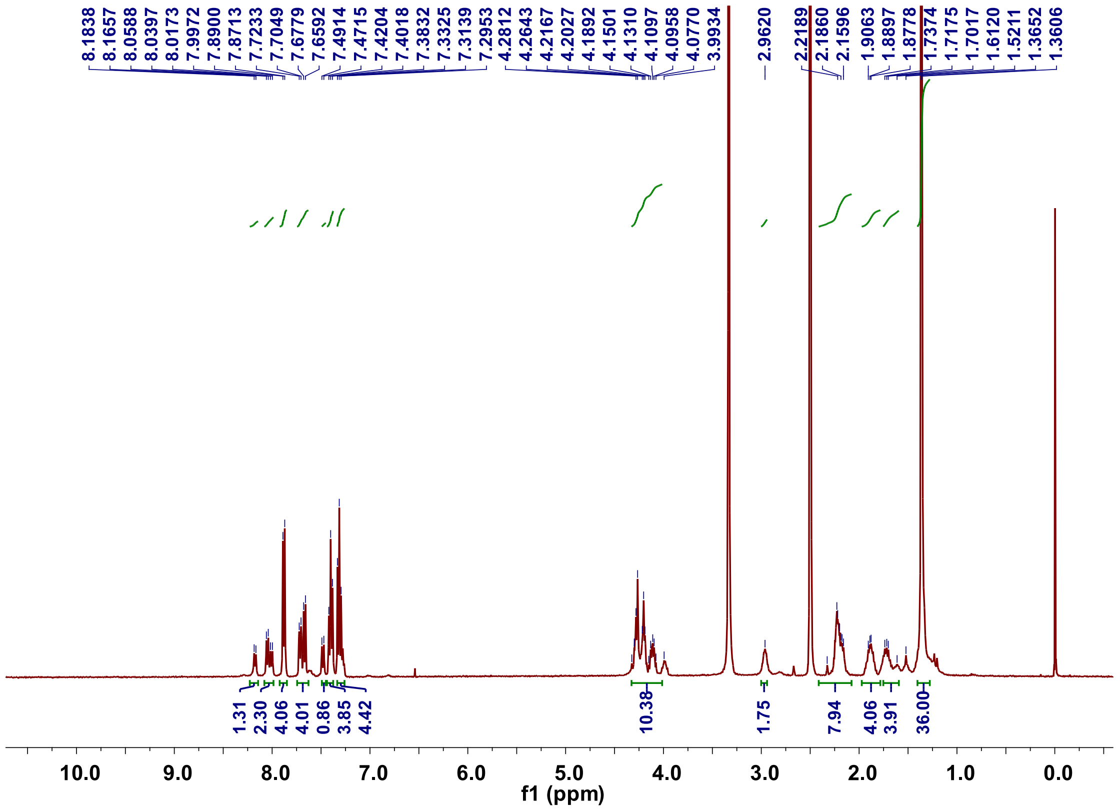

Figure S7. ^{1}H NMR spectra of ***Compound 2***. ^{1}H NMR (400 MHz, DMSO-d_6): δ 8.17 (d, *J* = 7.3 Hz, 1H), 8.03 (dd, *J* = 16.8, 7.9 Hz, 2H), 7.88 (d, *J* = 7.5 Hz, 4H), 7.69 (dd, *J* = 18.2, 7.4 Hz, 4H), 7.48 (d, *J* = 8.0 Hz, 1H), 7.40 (t, *J* = 7.5 Hz, 4H), 7.31 (t, *J* = 7.5 Hz, 4H), 4.35 – 3.96 (m, 10H), 2.96 (s, 2H), 2.35 – 2.12 (m, 8H), 1.96 – 1.82 (m, 4H), 1.78 – 1.51 (m, 4H), 1.36 (d, J = 1.8 Hz, 36H).

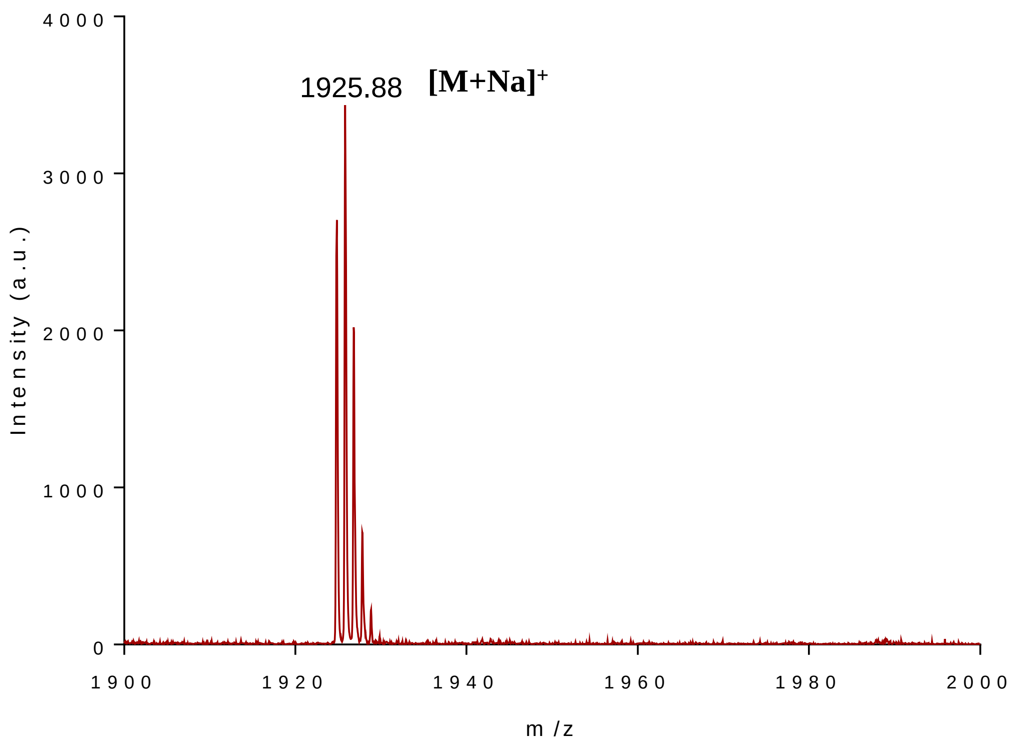

Figure S8. MALDI-TOF MS of ***Compound 3*** (m/z, $[M+Na]^+$): 1924.95 (calculated), 1925.88 (found).

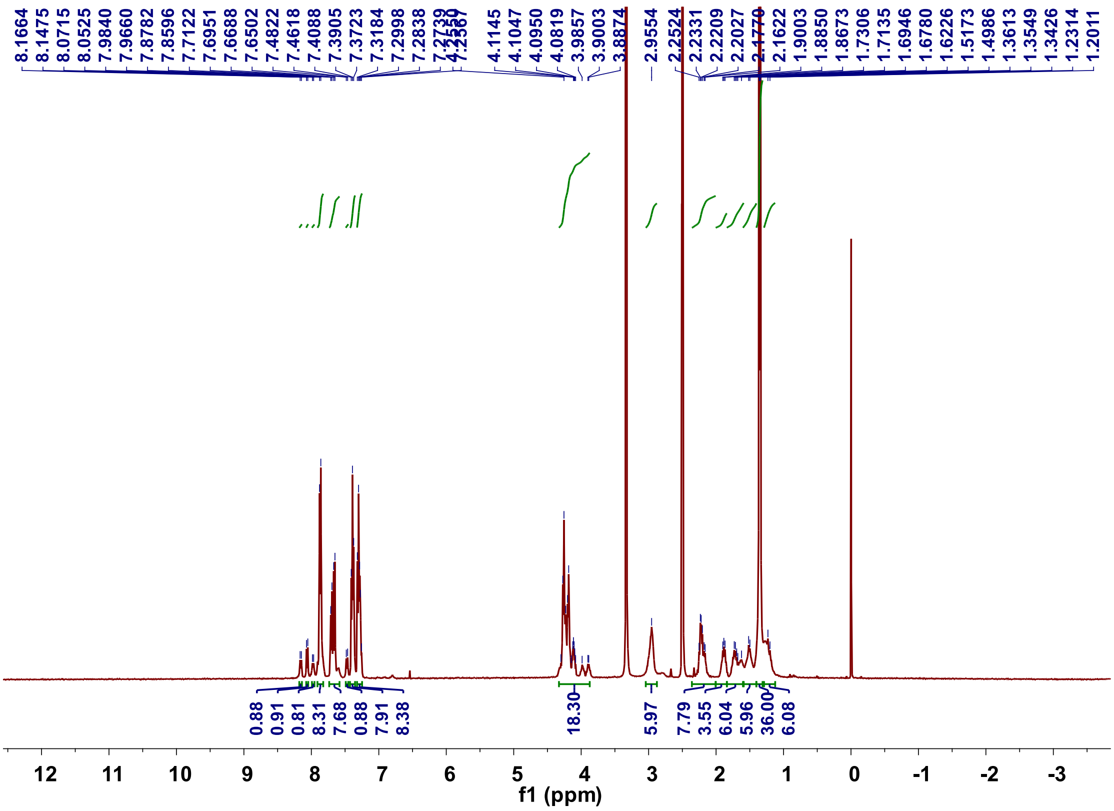

Figure S9. ^{1}H-NMR spectra of ***Compound 3***. ^{1}H NMR (400 MHz, DMSO-d_6): δ 8.16 (d, J = 7.5 Hz, 1H), 8.06 (d, J = 7.6 Hz, 1H), 7.98 (d, J = 7.2 Hz, 1H), 7.87 (d, J = 7.5 Hz, 8H), 7.68 (dd, J = 17.7, 7.1 Hz, 8H), 7.47 (d, J = 8.1 Hz, 1H), 7.39 (t, J = 7.3 Hz, 8H), 7.32 – 7.26 (m, 8H), 4.31 – 3.84 (m, 18H), 2.96 (s, 6H), 2.21 (m, 8H), 1.94 – 1.81 (m, 4H), 1.77 – 1.59 (m, 6H), 1.51 (d, J = 7.5 Hz, 6H), 1.46 – 1.30 (m, 36H), 1.22 (m, 6H).

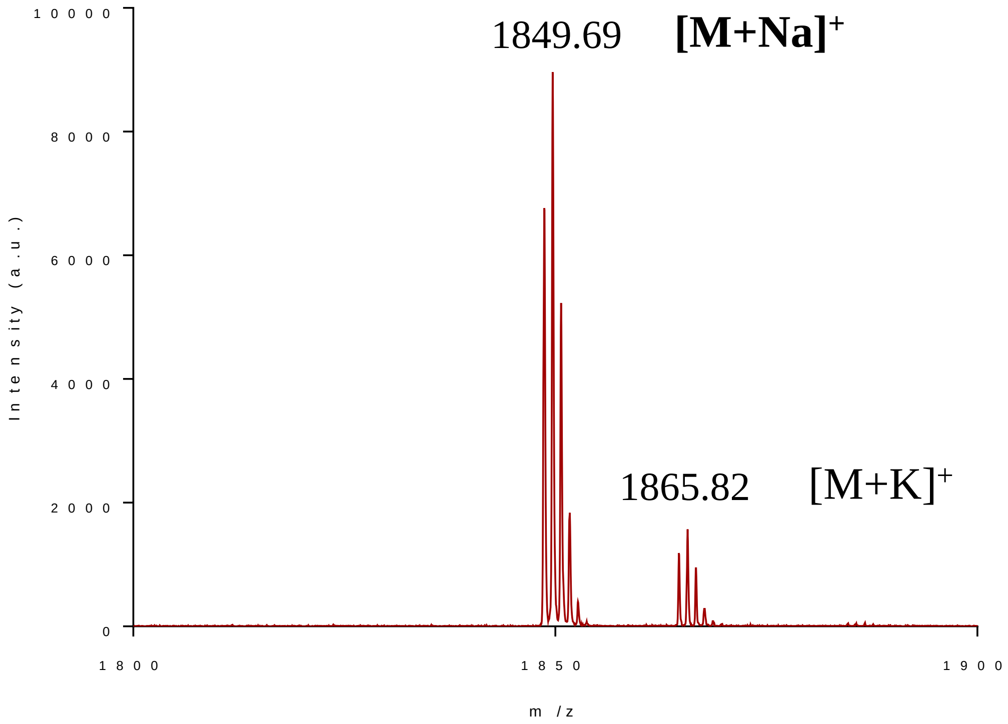

Figure S10. MALDI-TOF MS of ***Compound 4*** (m/z, [M+Na]$^+$): 1848.82 (calculated), 1849.69 (found).

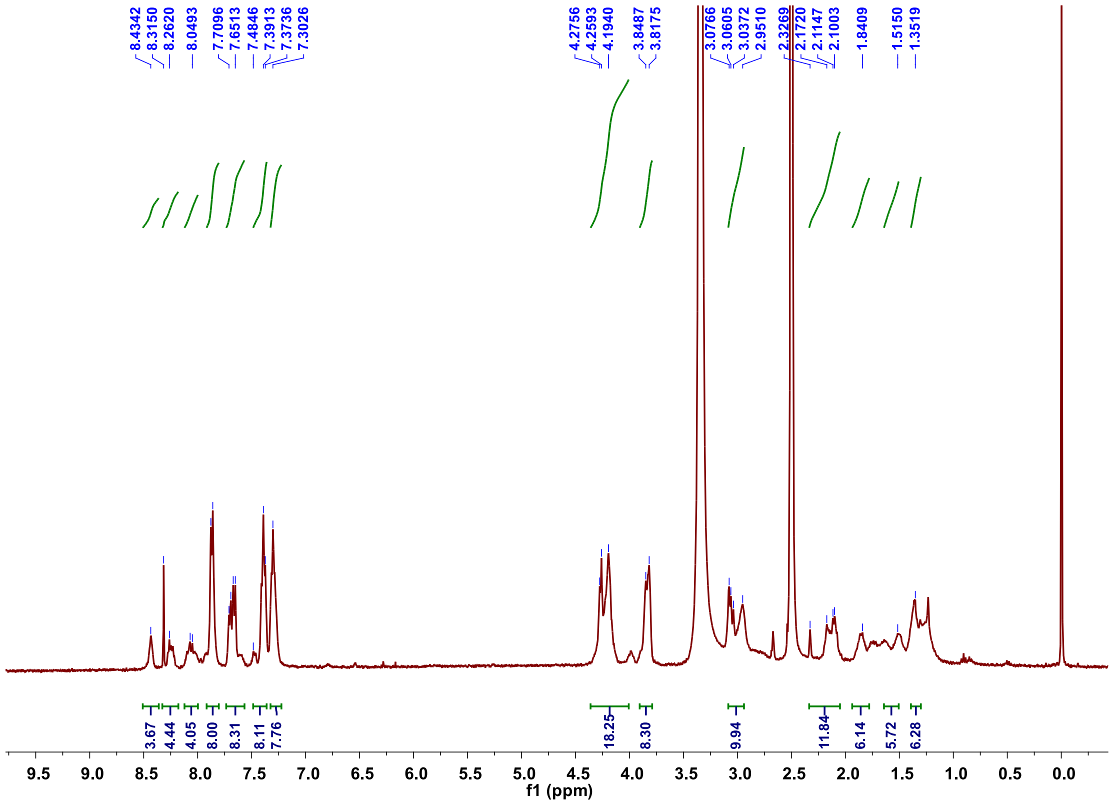

Figure S11. ^{1}H NMR spectra of ***Compound 4*** in DMSO-d_6. ^{1}H NMR (400 MHz, DMSO- d_6): δ 8.43 (s, 4H), 8.29 (m, 4H), 8.06 (d, *J* = 8.1 Hz, 4H), 7.87 (d, *J* = 7.1 Hz, 8H), 7.68 (dd, *J* = 16.0, 7.4 Hz, 8H), 7.38 (d, *J* = 7.1 Hz, 8H), 7.30 (s, 8H), 4.46-4.05 (m, 18H), 3.83 (d, *J* = 12.5 Hz, 8H), 3.03 (dd, *J* = 29.8, 20.5 Hz, 10H), 2.18 (m, 12H), 1.87-1.85 (m, 6H), 1.52 (s, 6H), 1.35 (s, 6H).

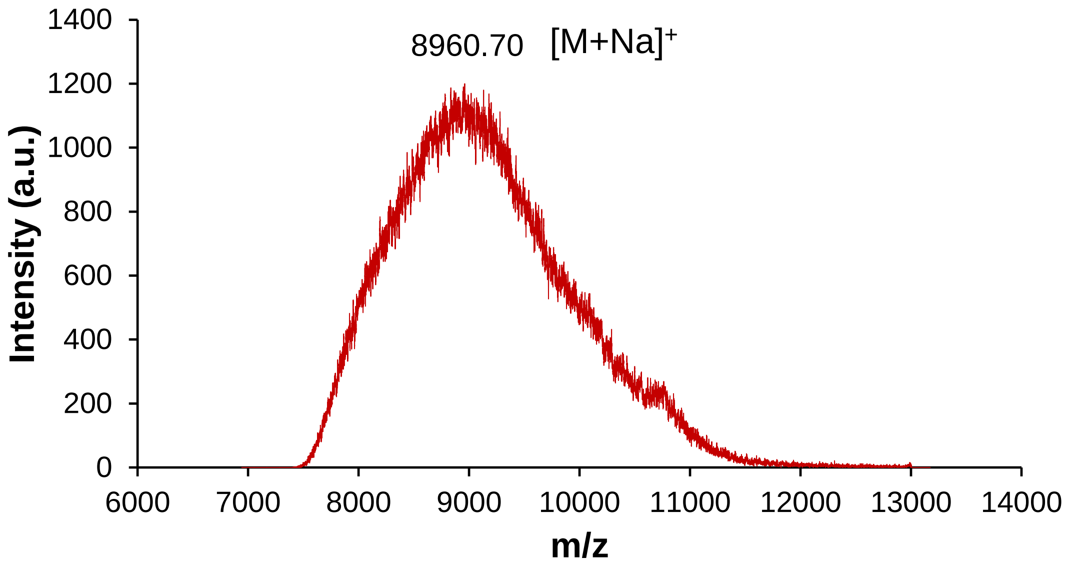

Figure S12. MALDI-TOF MS of ***Compound 5*** (m/z, $[M+Na]^+$): 8960.55 (calculated), 8960.70 (found).

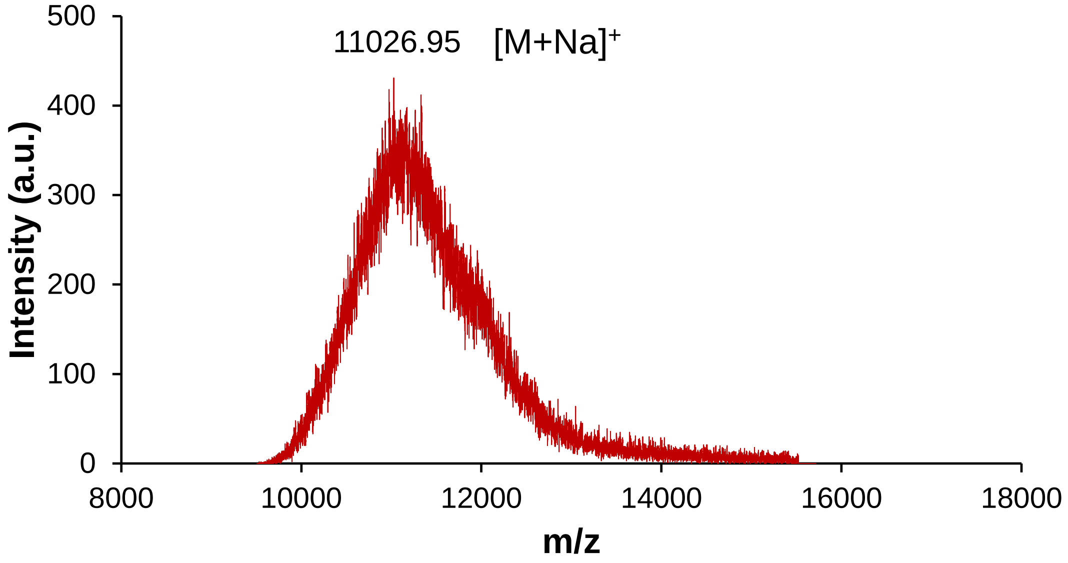

Figure S13. MALDI-TOF MS of ***PDPP*** (m/z, $[M+Na]^+$): 11027.15 (calculated), 11026.95 (found).

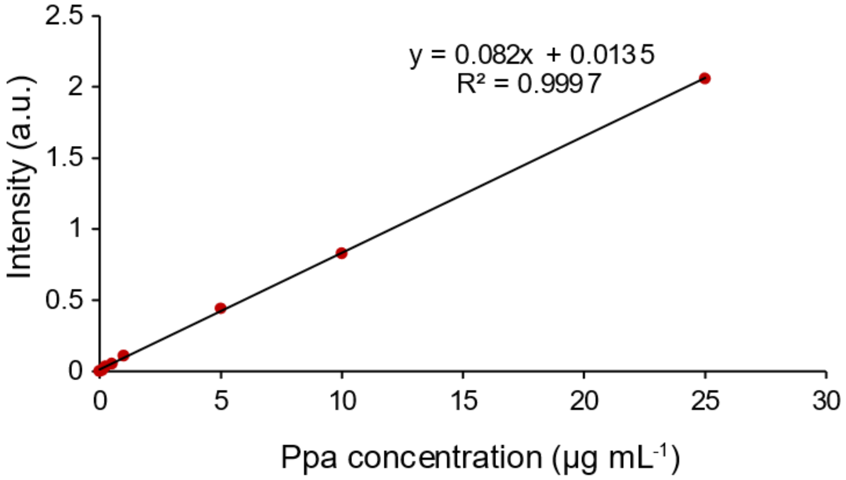

Figure S14. Standard absorption curve of Ppa in DMSO.

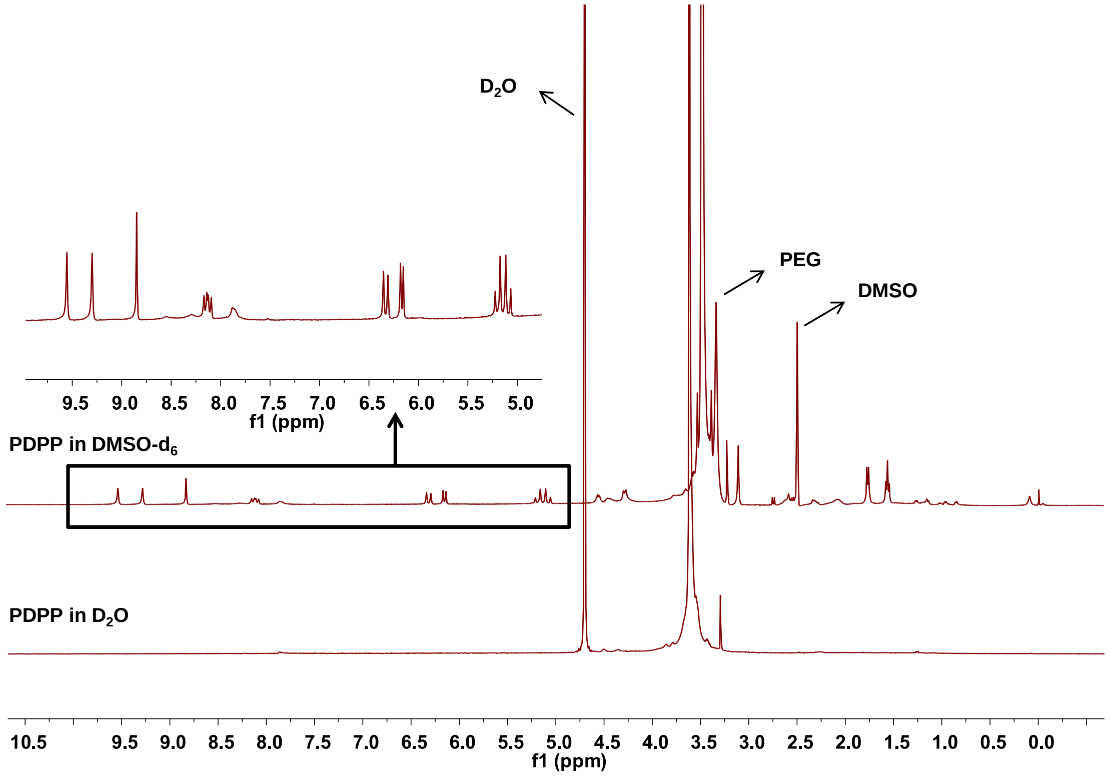

Figure S15. ^{1}H NMR spectra of PDPP in DMSO-d_6 and D_2O. After PDPP is dissolved in D_2O, the proton signals of Ppa completely disappear, implying occurrence of the self-assembly process of PDPP.

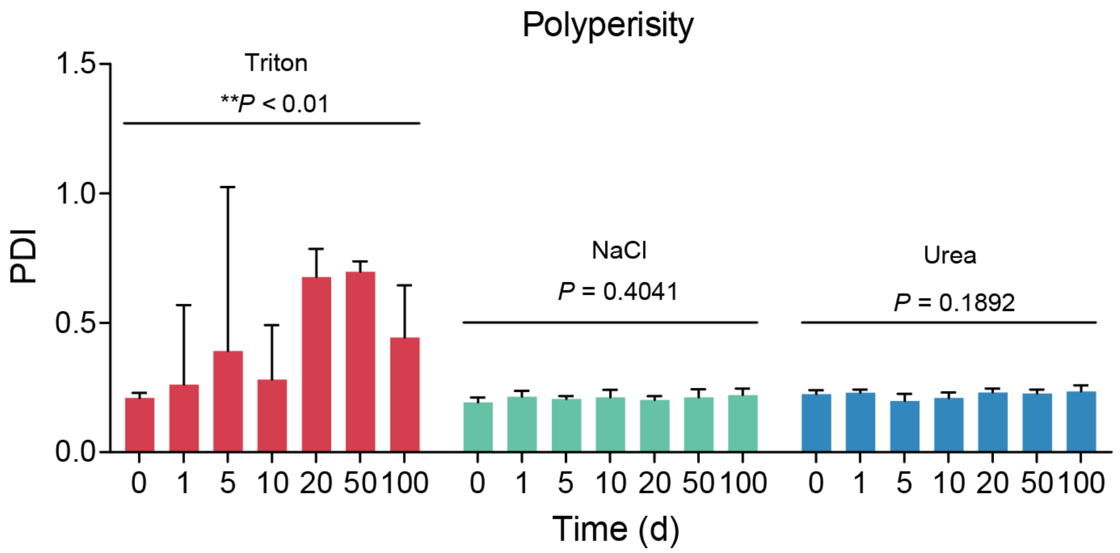

Figure S16. Changes in the PDI of PDPP after it is dispersed in Triton, NaCl and urea solutions.

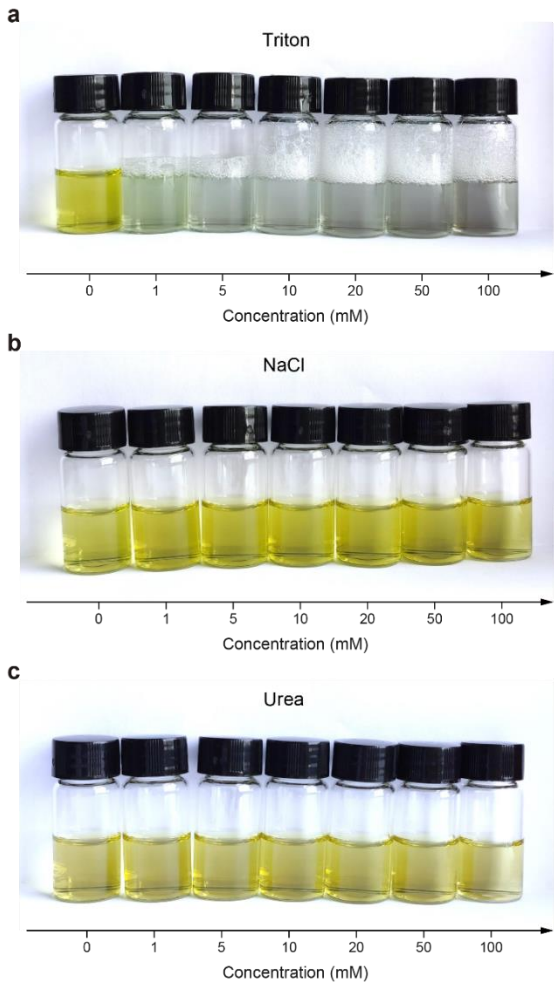

Figure S17. Photographic images of 0.5 mg mL^{-1} PDPP dispersed in different aqueous solution containing Triton (a), NaCl (b) and urea (c) at different concentrations.

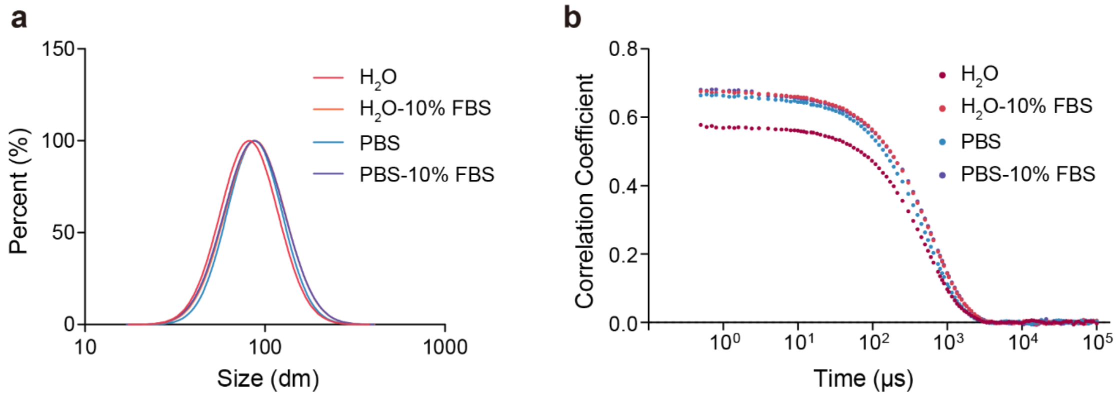

Figure S18. Size distribution a) and correlation coefficient (b) of PDPP in different solutions.

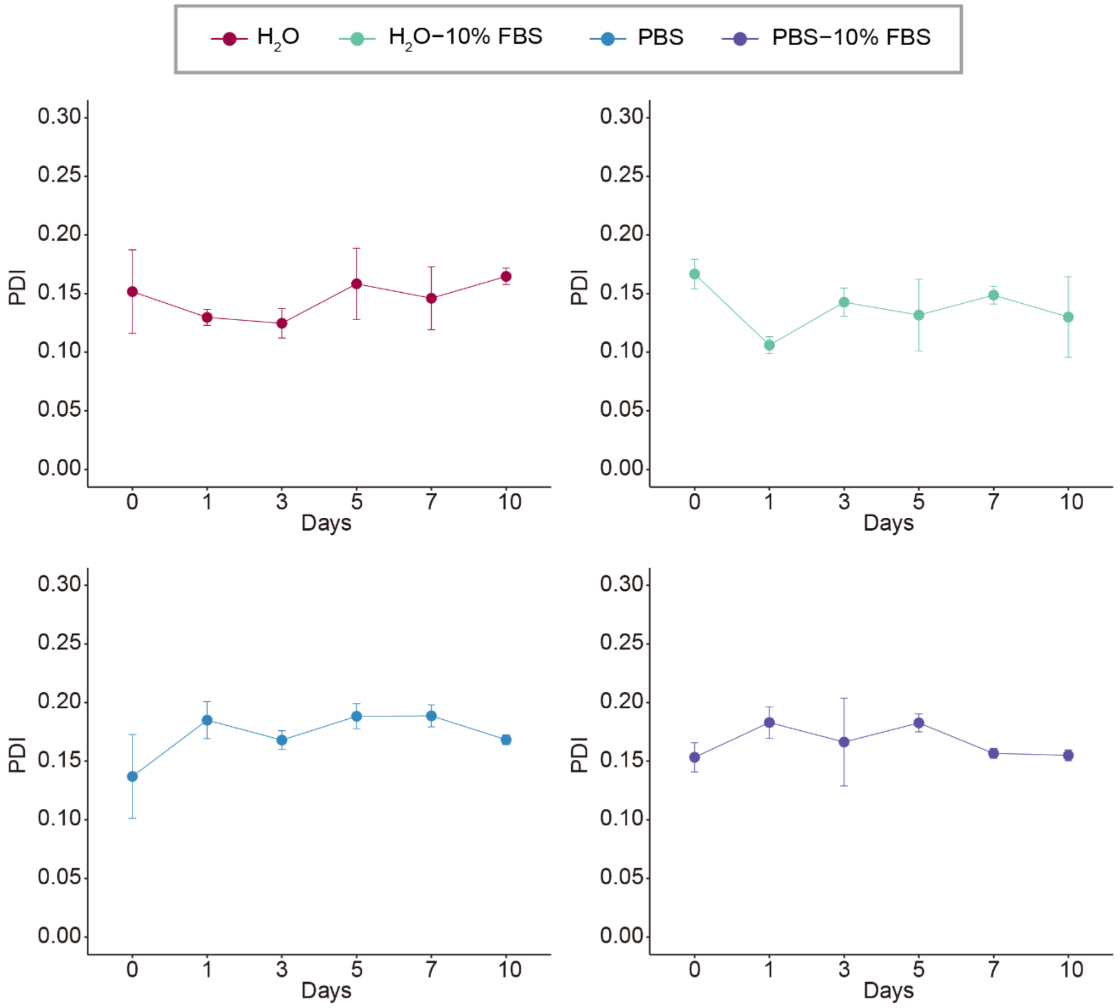

Figure S19. Colloidal stability of PDPP in different solutions at 37°C (n = 3) from day 0 to day 10. All the measured data of PDI are less than 0.3.

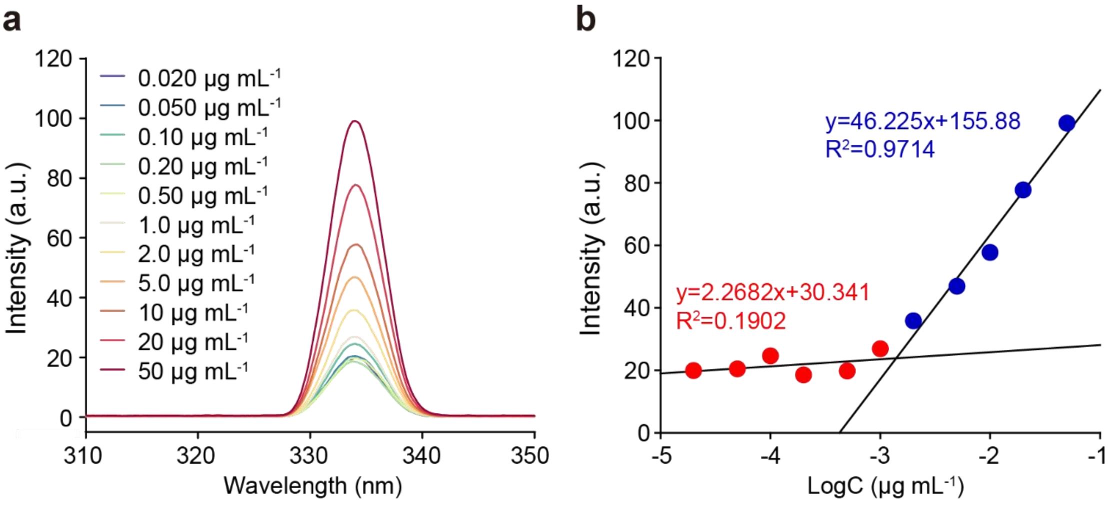

Figure S20. The fluorescence intensity of the fluorescent probe pyrene in PDPP at different concentrations (a) to determine CAC of PDPP through a linear regression curve (b).

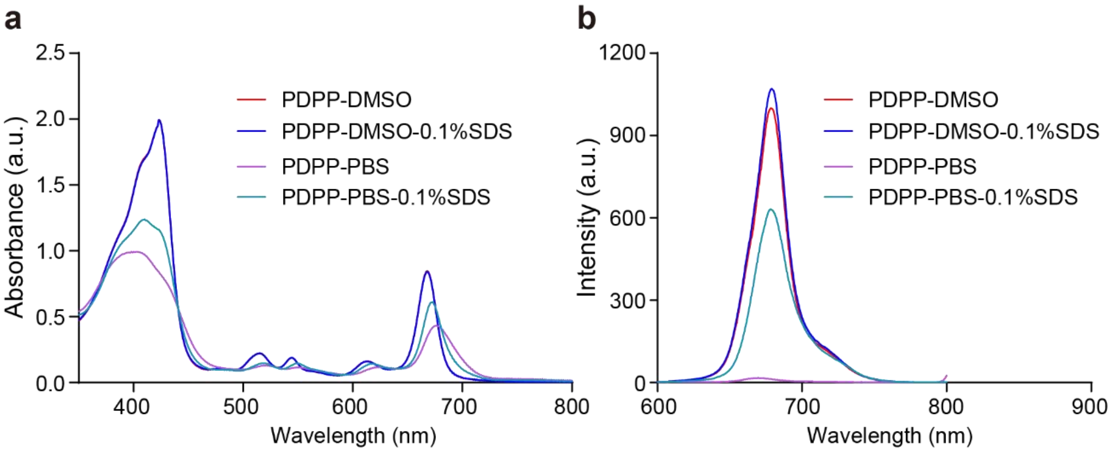

Figure S21. (a) UV-vis absorbance spectra of PDPP measured by different treatments. (b) Fluorescence spectra of PDPP in different solvents.

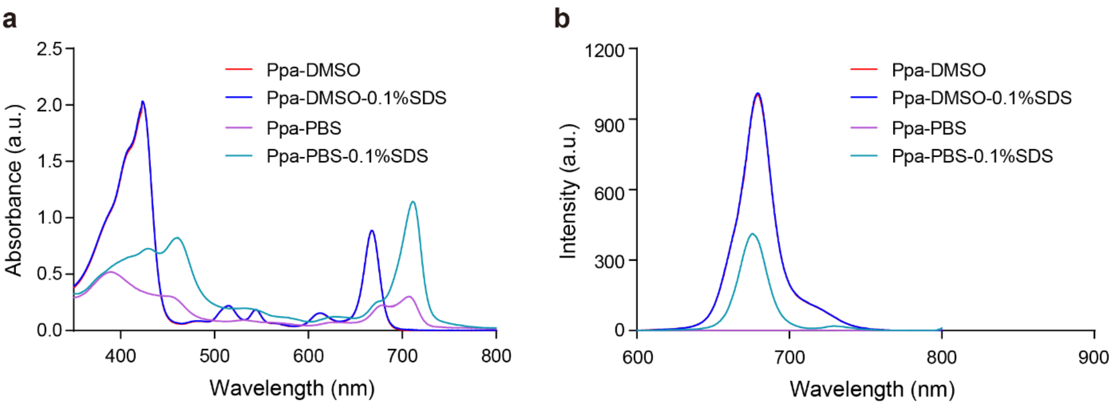

Figure S22. (a) UV-vis absorbance spectra of Ppa measured by different treatments. (b) Fluorescence spectra of Ppa in different solvents.

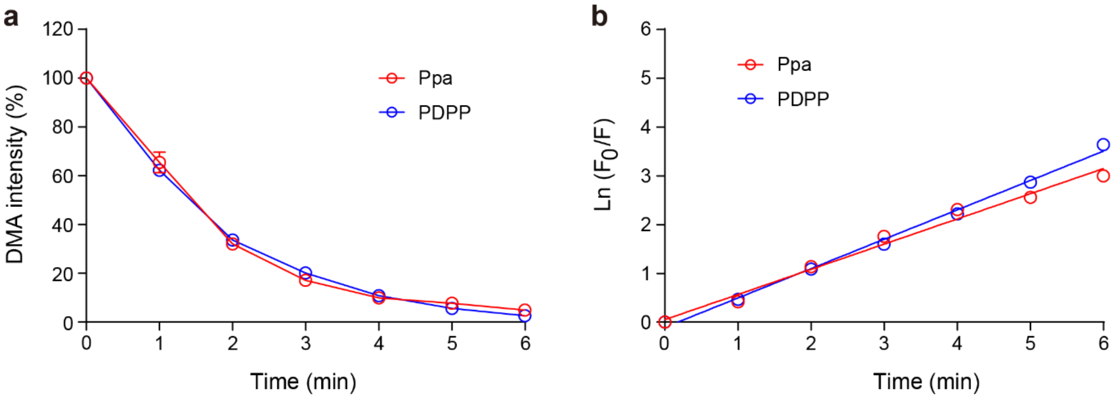

Figure S23. Singlet oxygen generation by PDPP and Ppa measured by DMA.

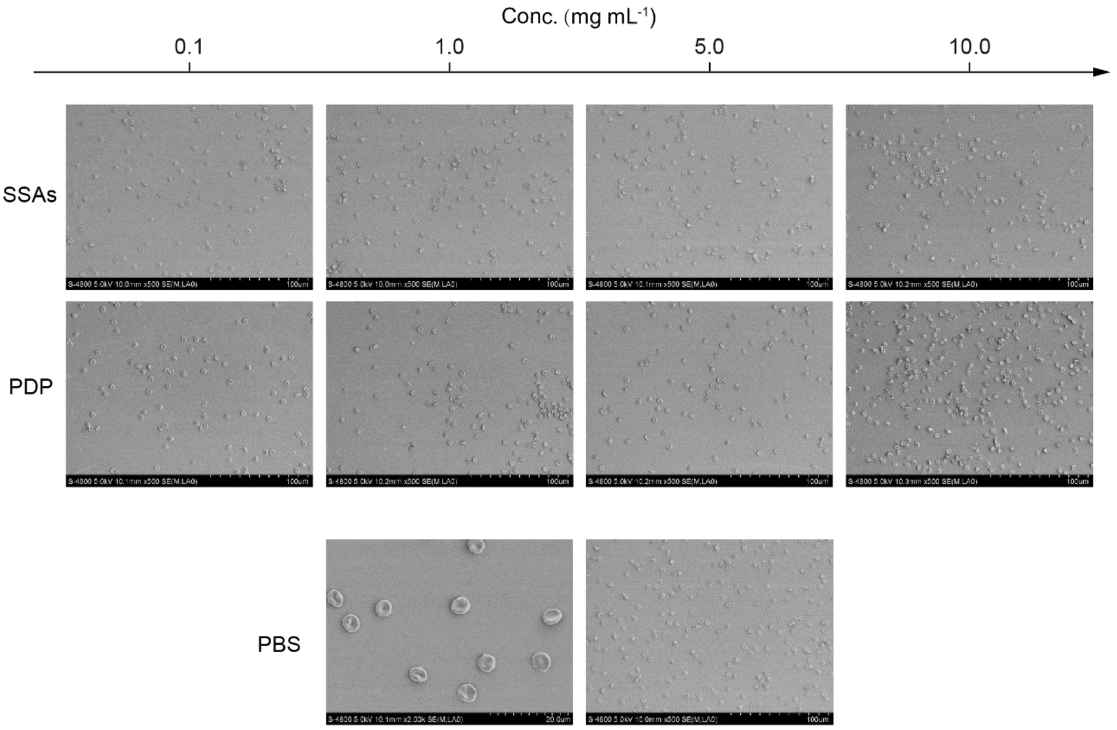

Figure S24. SEM images for revealing effects of PDP and SSAs at different concentrations on morphologies and aggregation of erythrocytes compared with RBCs with the PBS treatment as a control.

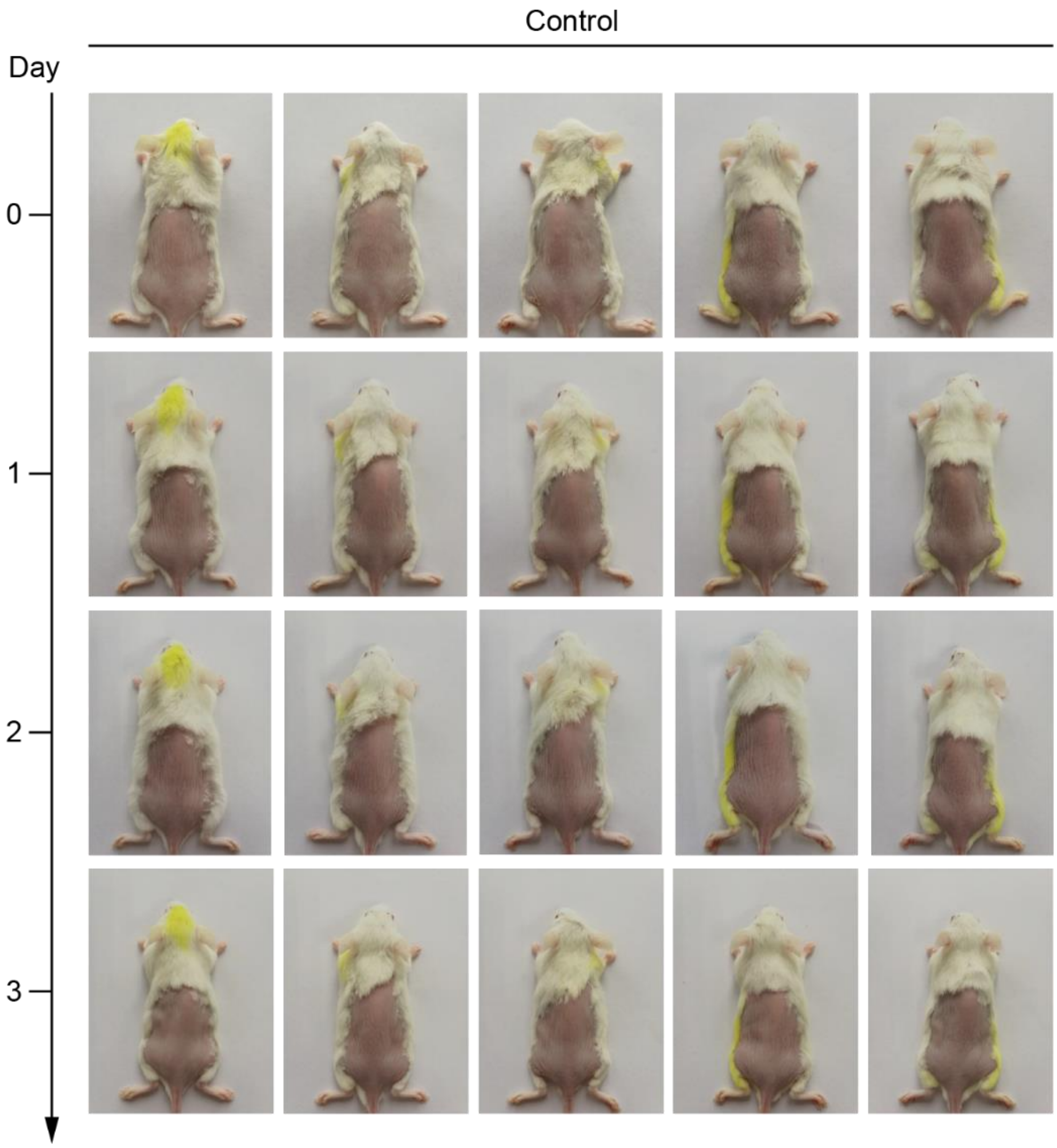

Figure S25. The shaved back of BALB/c mice (n=5) receiving intravenous injection of PBS as a control.

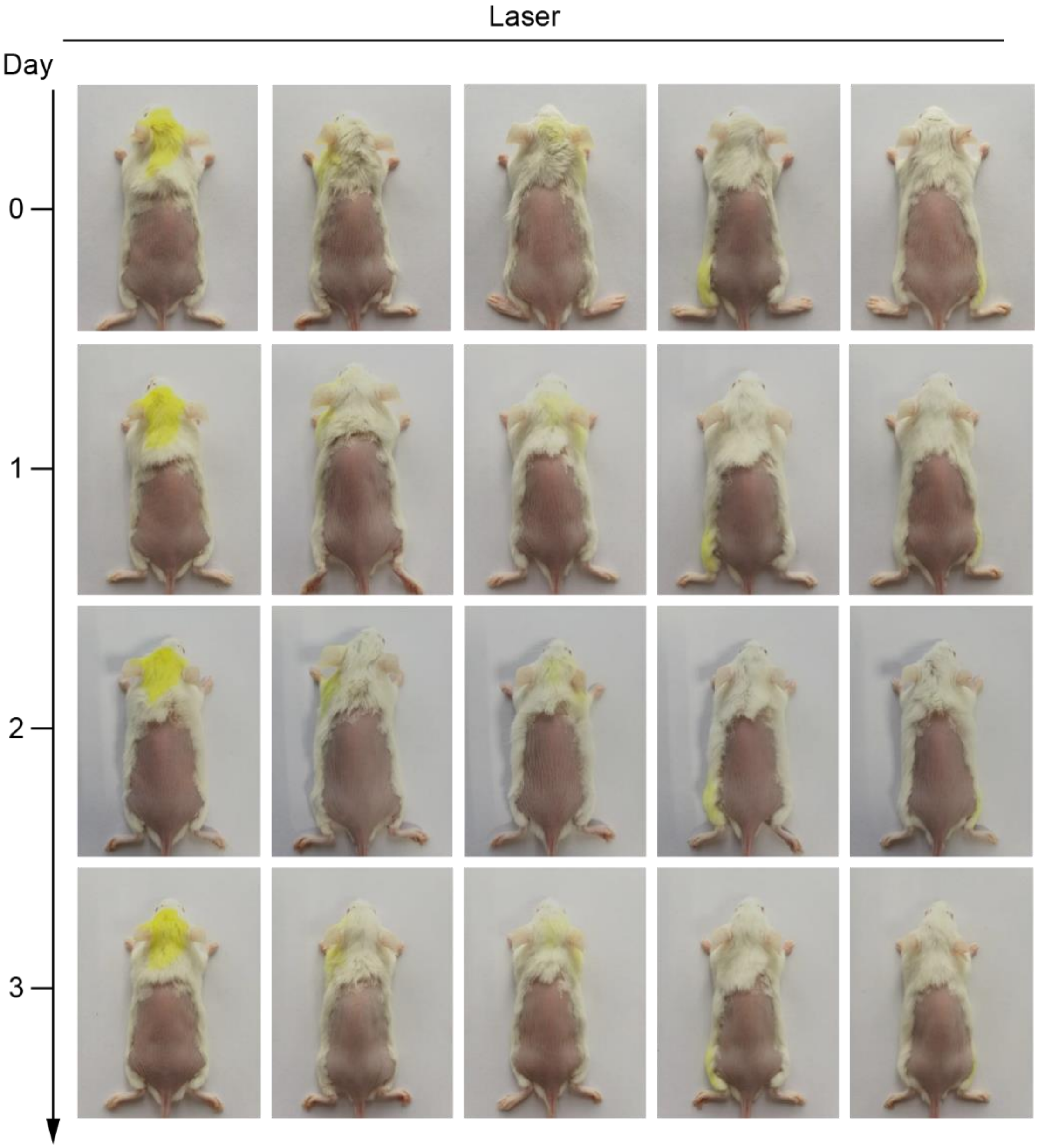

Figure S26. The shaved back of BALB/c mice (n=5) receiving irradiation of a 660 nm laser (10 min, 108 J cm^{-2}).

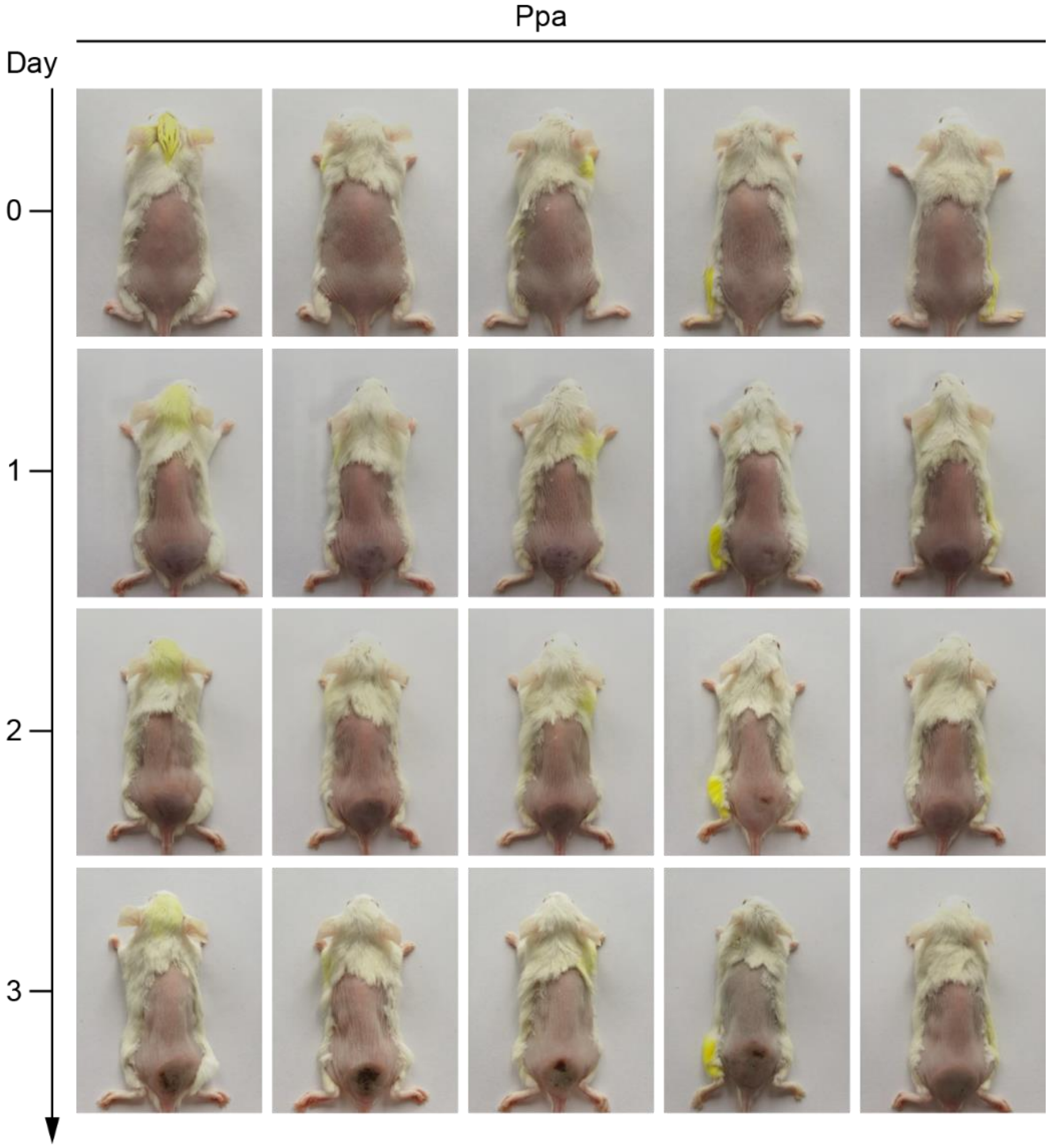

Figure S27. The shaved back of BALB/c mice (n=5) receiving intravenous injection of Ppa under irradiation of a 660 nm laser (10 min, 108 J cm^{-2}).

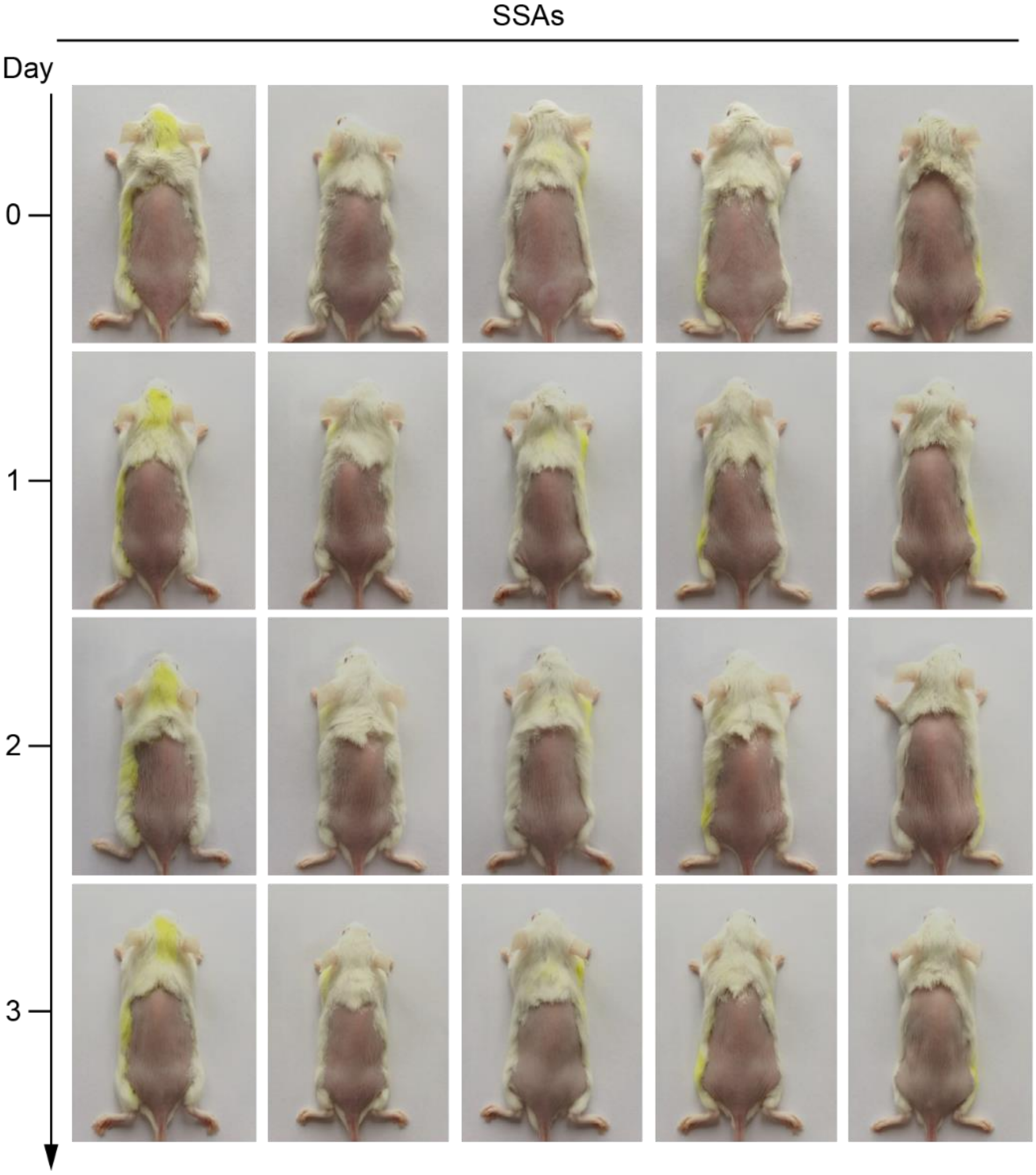

Figure S28. The shaved back of BALB/c mice (n=5) receiving intravenous injection of SSAs under irradiation of a 660 nm laser (10 min, 108 J cm^{-2}).

5

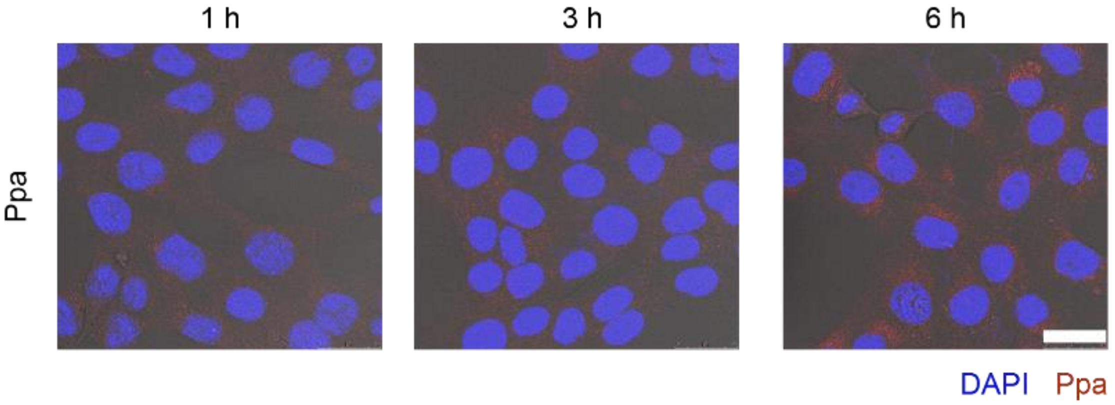

Figure S29. Representative fluorescence images of 4T1 cells after incubation with Ppa. Scale bar: 25 μm.

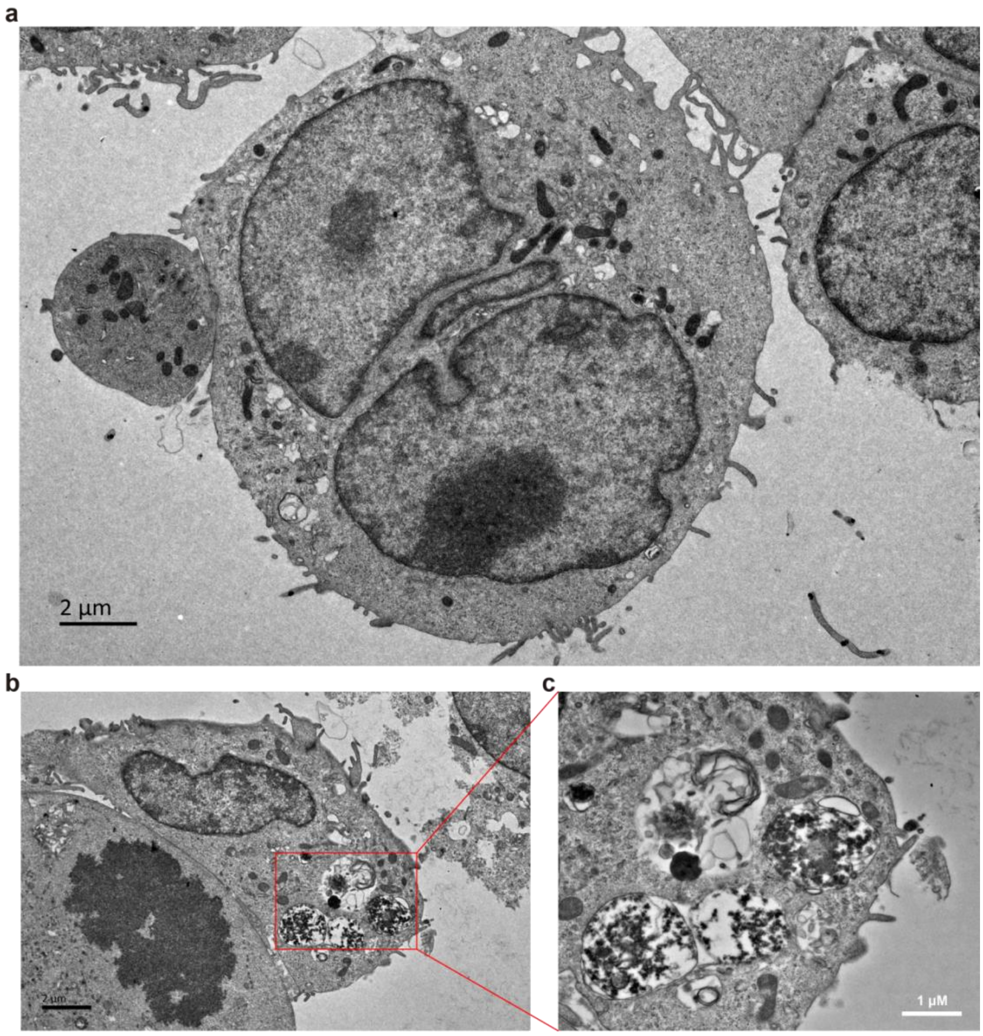

Figure S30. TEM images and its magnified views for the inner structure of 4T1 cells without treatment a) and after treatment with Ppa (b, c) for 24 h.

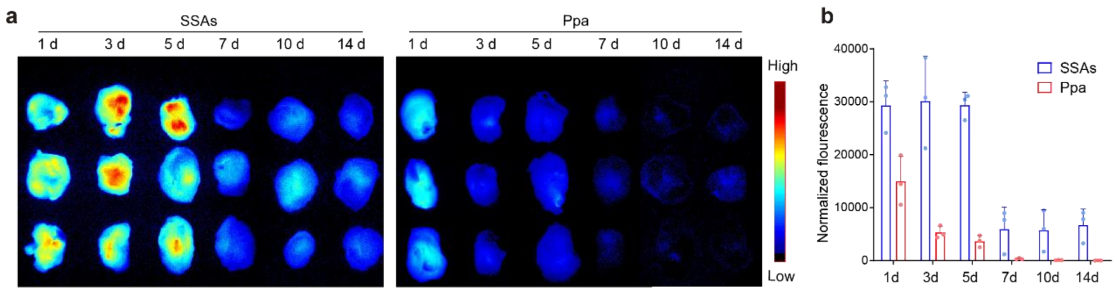

Figure S31. *Ex vivo* fluorescent imaging (a) and normalized fluorescence intensity (b) of tumor tissues excised from 4T1 tumor-bearing mice administrated with SSAs and Ppa at pre-determined time points. Data are presented as mean ± s.d., n = 3.

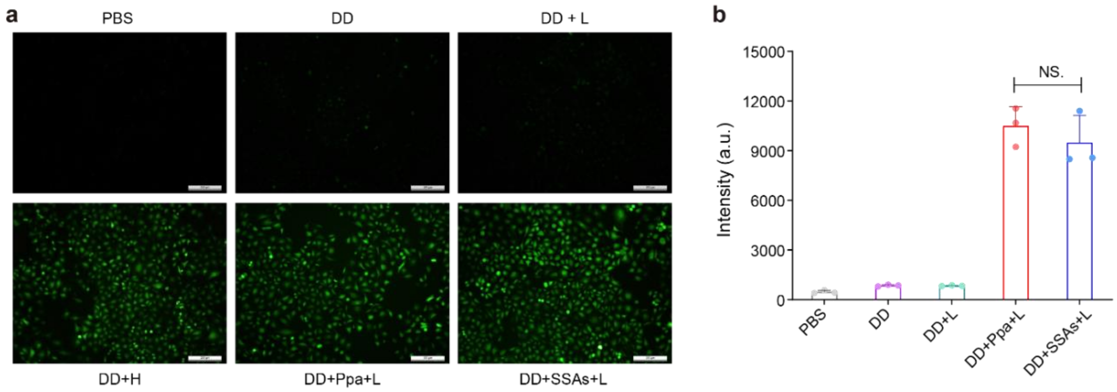

Figure S32. (a) ROS-generation in 4T1 cells exposed to different treatments using a fluorescence probe DCFH-DA (1 J cm^{-2}, 660 nm; DD: DCFH-DA; L: laser; H: H_2O_2); Green fluorescence for positive staining of ROS, scale bar: 200 μm. (b) ROS-generation in 4T1 cells from flow cytometry (Data are presented as mean ± s.d., n=3).

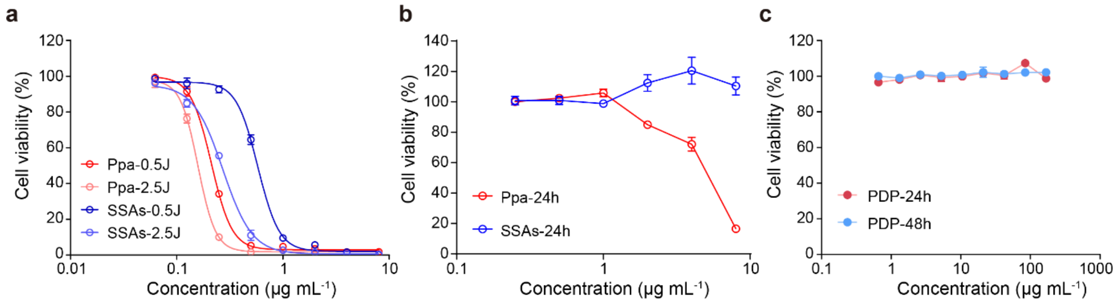

Figure S33. Cytotoxicity of SSAs and Ppa against 4T1 cells with (a) and without (b) laser irradiation (n=3); (c) Cytotoxicity of PDP against L02 cells without laser irradiation (n=3).

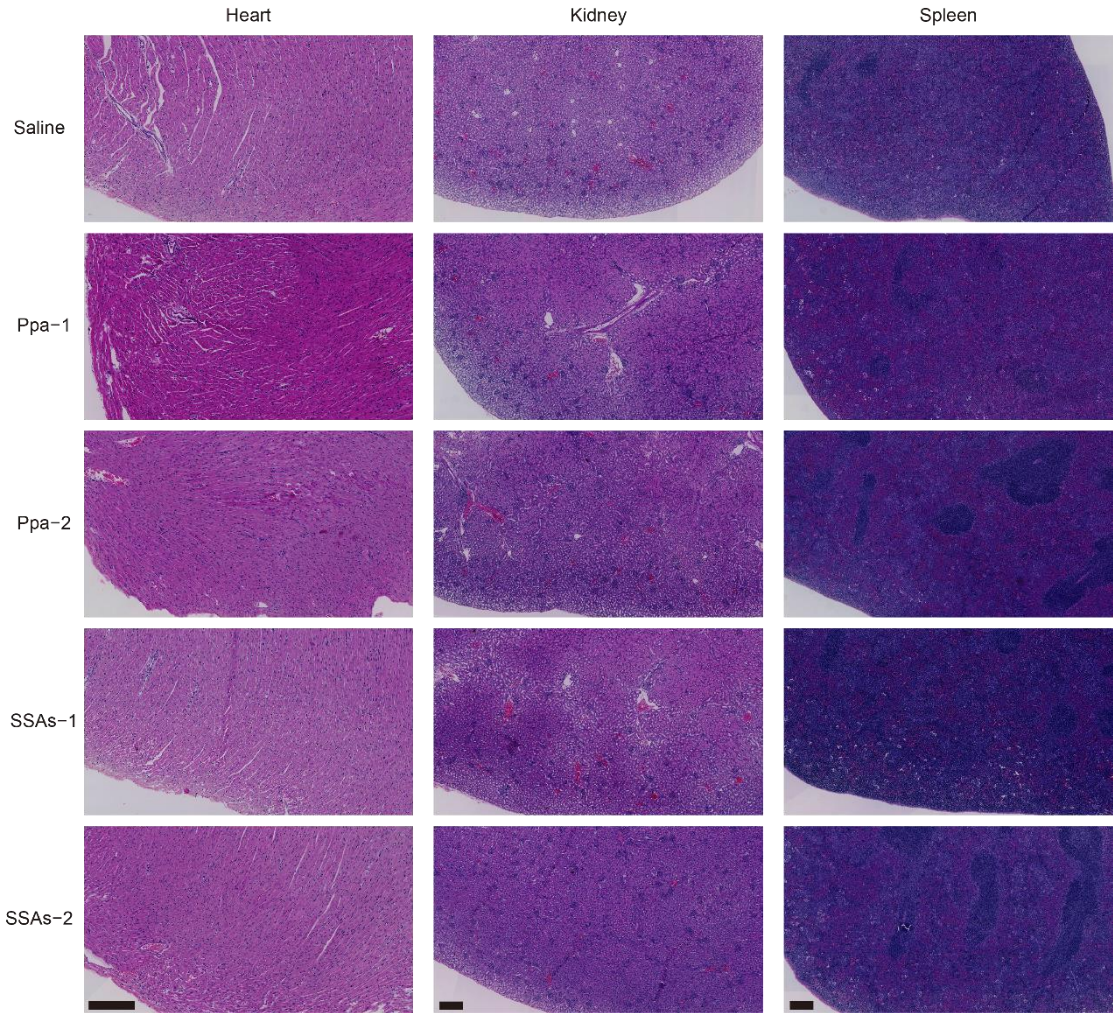

Figure S34. H&E staining images for the heart, kidney and spleen harvested from tumor-bearing mice receiving different treatments. Scale bar: 250 μm.

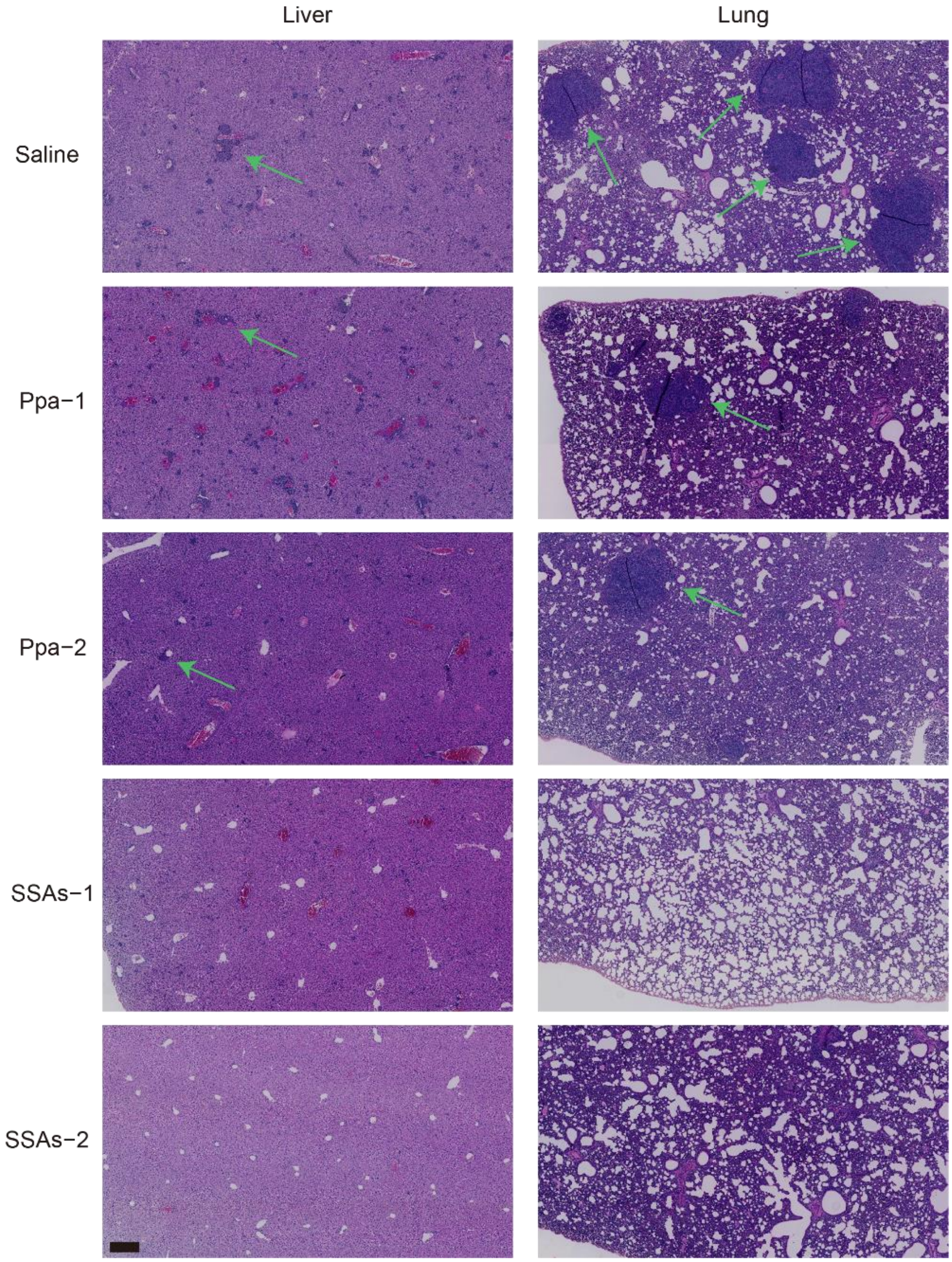

Figure S35. H&E staining images for the liver and lung harvested from tumor-bearing mice receiving different treatments. Arrows indicate the metastatic lesions of tumors. Scale bar: 250 μm.

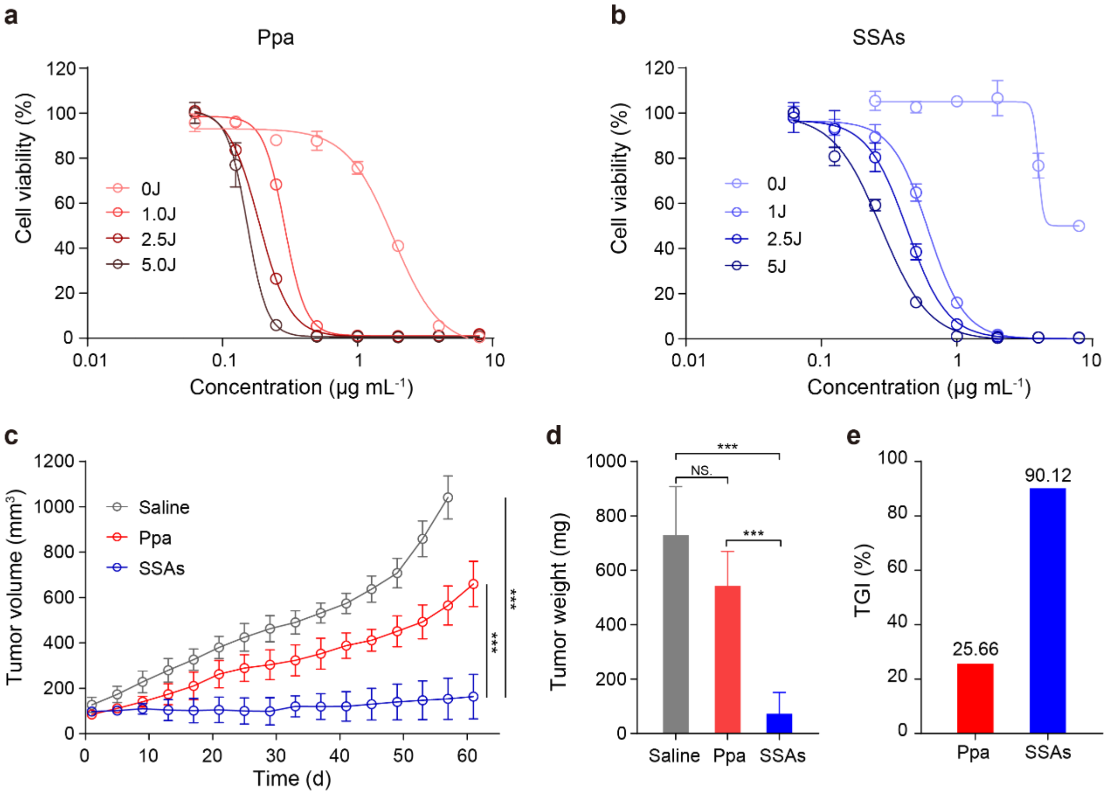

Figure S36. (a, b) Cytotoxicity of Ppa (a) and SSAs (b) against A549 cells at different doses of laser irradiation. (c) *In vivo* PDT efficacy of SSAs against subcutaneous A549 tumors in nude mice and the averaged A549 tumor volume curves after different treatments (n=5). (d, e) Tumor weights (d) and calculated TGIs (e) according to the tumor weights on Day 61.

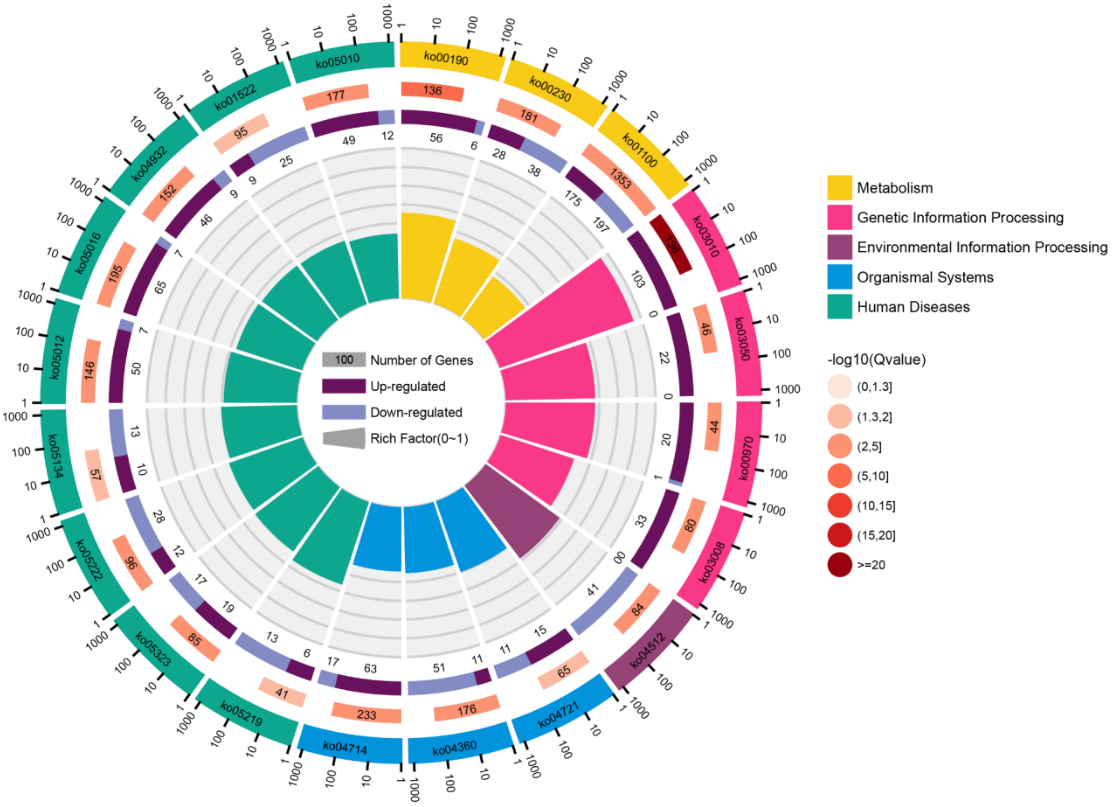

Figure S37. A circos image with top 20 terms of enriched pathways after KEGG pathway enrichment analysis of significantly regulated genes from 4T1 cells treated by Ppa.

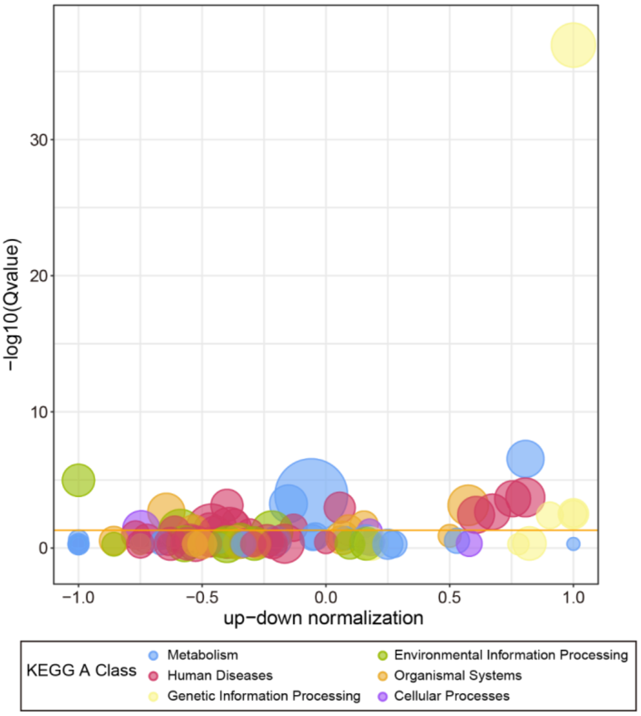

Pathway Top 20

ID	Descrption
ko00190	Oxidative phosphorylation
ko01100	Metabolic pathways
ko00230	Purine metabolism
ko04512	ECM−receptor interaction
ko05016	Huntington disease
ko05012	Parkinson disease
ko05222	Small cell lung cancer
ko05323	Rheumatoid arthritis
ko04932	Non−alcoholic fatty liver disease (NAFLD)
ko05010	Alzheimer disease
ko05219	Bladder cancer
ko05134	Legionellosis
ko01522	Endocrine resistance
ko04714	Thermogenesis
ko04360	Axon guidance
ko04721	Synaptic vesicle cycle
ko03010	Ribosome
ko03008	Ribosome biogenesis in eukaryotes
ko03050	Proteasome
ko00970	Aminoacyl−tRNA biosynthesis

Figure S38. KEGG pathway enrichment analysis of significantly regulated genes from

4T1 cells treated by Ppa. Bubbles in the left graph show the top terms of enriched pathways, and the bubble size corresponds to the scores of up/down-regulation that are normalized from 1 to -1. Detailed pathways with different colors are presented in the right graph.

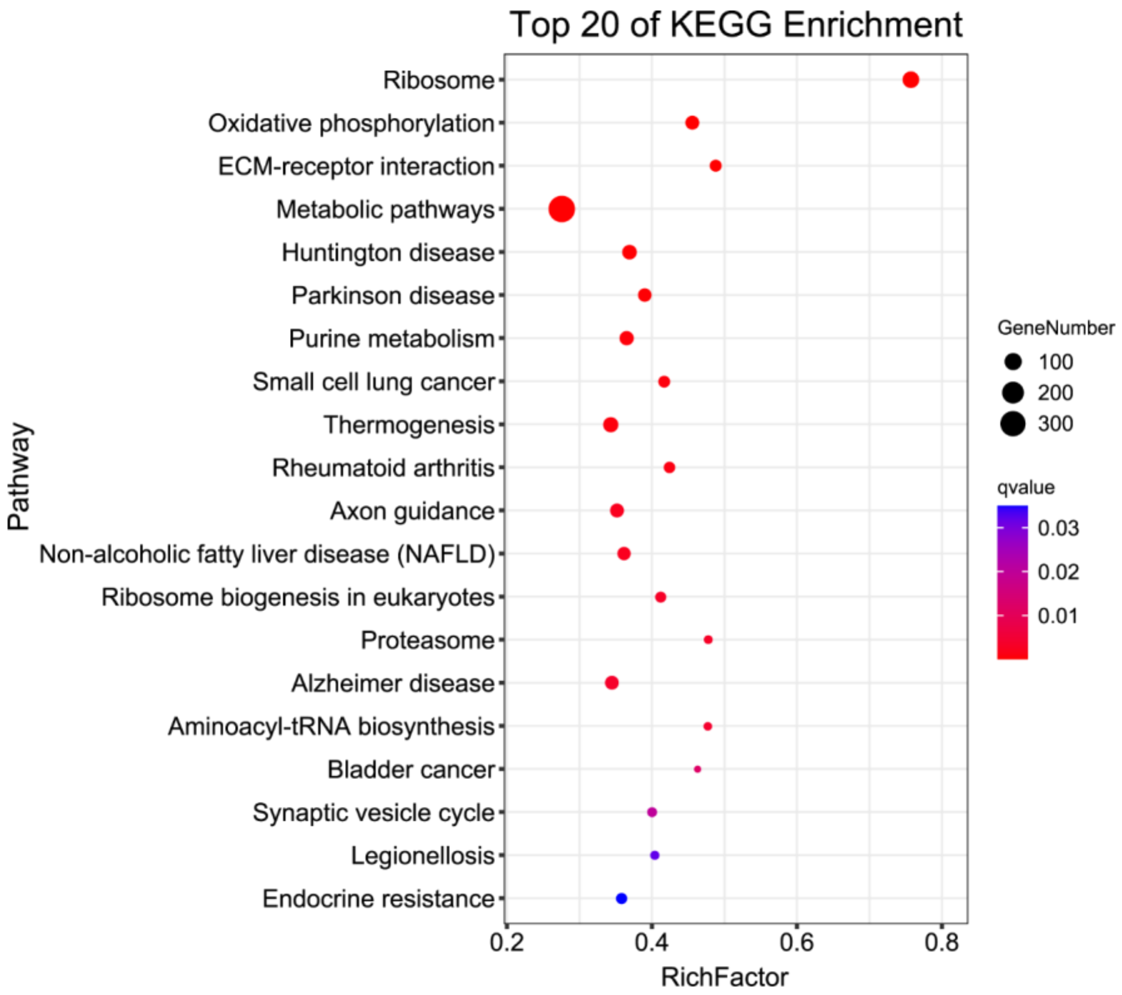

Figure S39. Significant abundance changes of expressed genes (*P* value < 0.05) in samples treated by Ppa via the KEGG pathway enrichment analysis. Top 20 terms of regulated KEGG pathway enrichment in an order of -log10 (Q value) are shown.

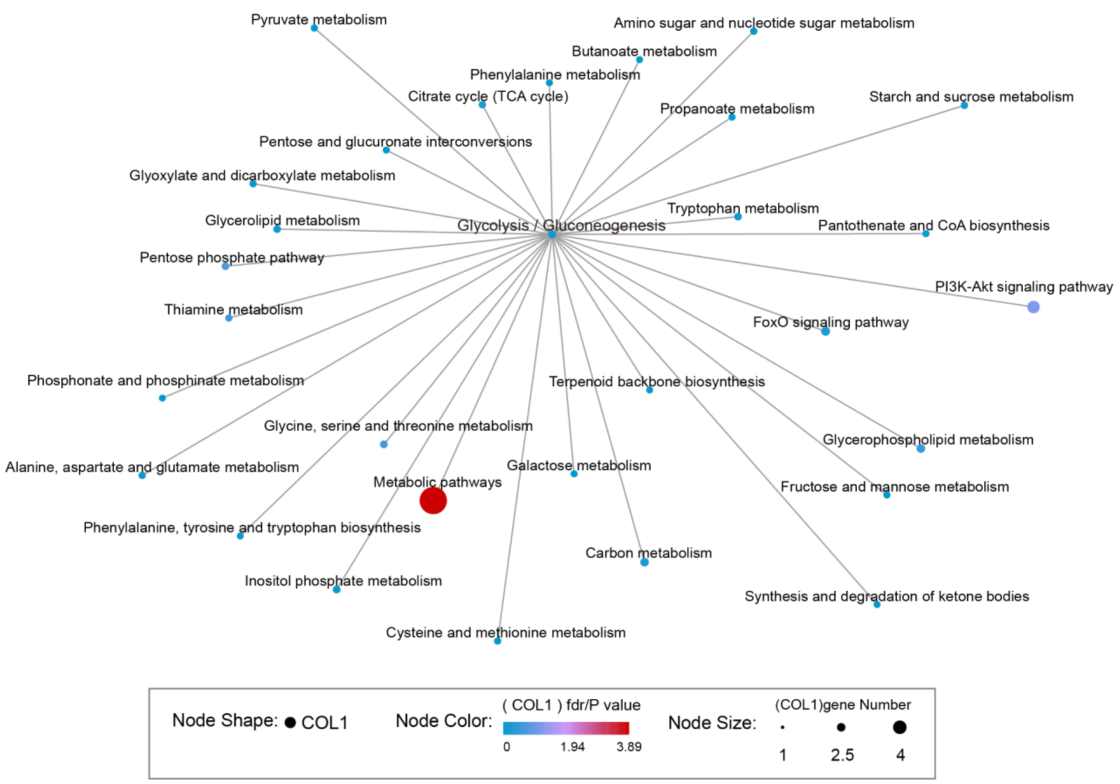

Figure S40. Interaction networks of enriched KEGG pathways from treated 4T1 cells by Ppa. This result indicates the PI3K/Akt pathway regulated by Ppa interacts with metabolic pathways.

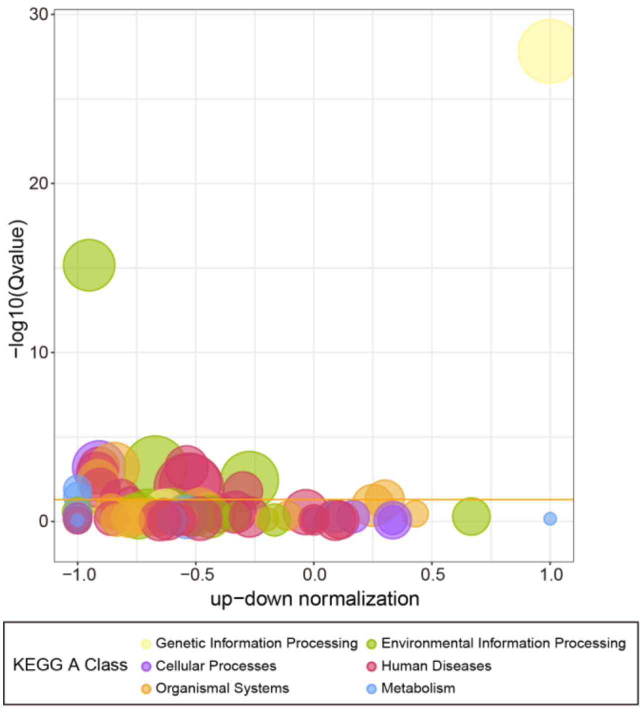

Pathway Top 20

ID	Descrption
ko00100	Steroid biosynthesis
ko00900	Terpenoid backbone biosynthesis
ko04512	ECM−receptor interaction
ko04151	PI3K−Akt signaling pathway
ko04010	MAPK signaling pathway
ko04510	Focal adhesion
ko05222	Small cell lung cancer
ko05412	Arrhythmogenic right ventricular cardiomyopathy (ARVC)
ko05414	Dilated cardiomyopathy (DCM)
ko05165	Human papillomavirus infection
ko05410	Hypertrophic cardiomyopathy (HCM)
ko05200	Pathways in cancer
ko05323	Rheumatoid arthritis
ko05146	Amoebiasis
ko01523	Antifolate resistance
ko04360	Axon guidance
ko04974	Protein digestion and absorption
ko04657	IL−17 signaling pathway
ko04380	Osteoclast differentiation
ko03010	Ribosome

Figure S41. KEGG pathway enrichment analysis of significantly regulated genes from 4T1 cells treated by SSAs. Bubbles in the left graph show the top terms of enriched

pathways, and the bubble size corresponds to the scores of up/down-regulation that are normalized from 1 to -1. Detailed pathways with different colors are presented in the right graph.

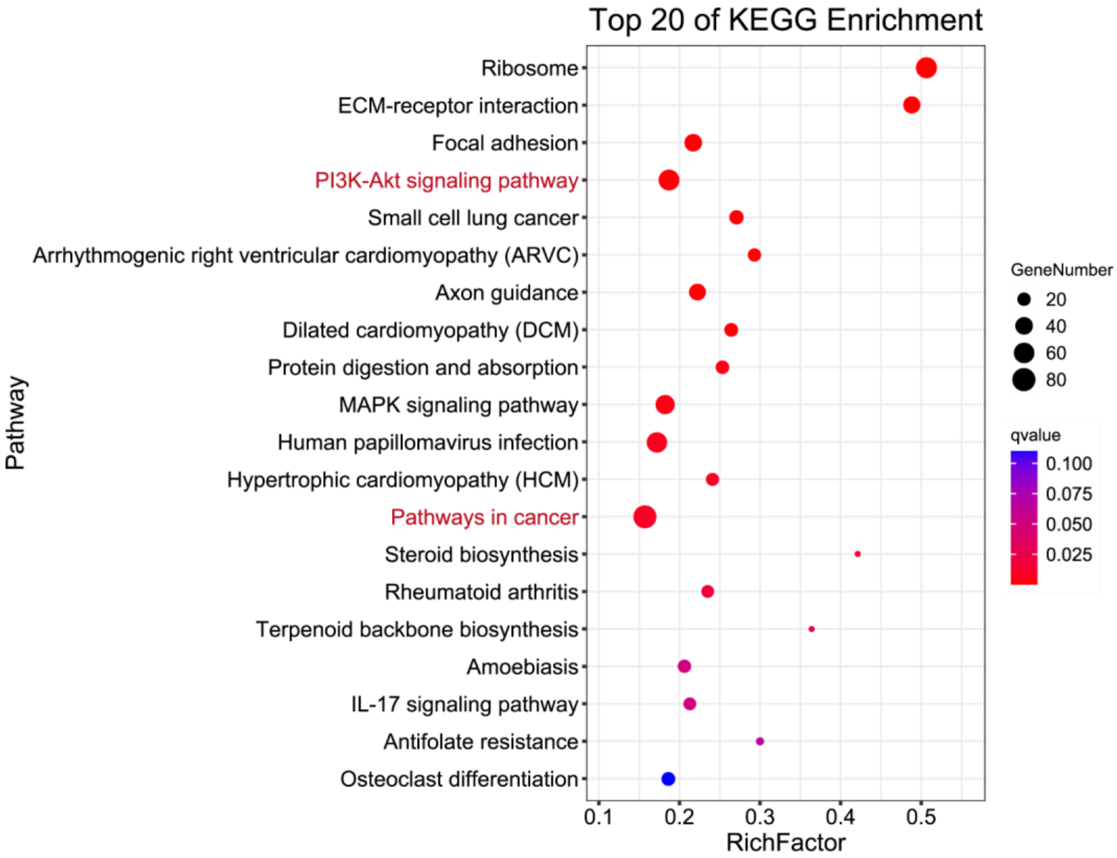

Figure S42. Significant abundance changes of expressed genes (*P* value < 0.05) in samples treated by SSAs via the KEGG pathway enrichment analysis. Top 20 terms of regulated KEGG pathway enrichment in an order of -log10 (Q value) are shown.

Table S1. Interaction parameters between different beads.

α_{ij}	E	G	L	P	H
E	25	25	27	37	31
G	-	25	26	34	33
L	-	-	25	30	38
P	-	-	-	25	59
H	-	-	-	-	25

Table S2. Size, zeta potential and PDI of PDPP in different solutions.

Solutions	Size (d.nm)	Zeta Potential (mV)	PDI
H_2O	104.68±15.10	-25.49±0.58	0.152±0.036
PBS	100.38±19.12	-5.28±7.86	0.137±0.036
H_2O+10%FBS	96.13±9.72	-13.57±0.85	0.167±0.012
PBS+10%FBS	102.58±8.94	-7.34±0.94	0.154±0.013

Table S3. Photophysical characterizations of PDPP and Ppa.

Groups	SOQ	ε /10^4 $M^{-1}\cdot cm^{-1}$	FQ
PDPP	0.51	3.07	0.28
Ppa	0.50	3.49	0.30

a) SOQ (singlet oxygen quantum yield ($\Phi_{\triangle}$)) in DMSO; b) Molar extinction coefficients (ε) in DMSO; and c) FQ (Fluorescent quantum yield ($\Phi_{\triangle}$)) in toluene.

Table S4. Pharmacokinetic parameters of SSAs and Ppa.

Groups	$t_{1/2}$ [a)] (h)	AUC [b)] ($mg\cdot L^{-1}\cdot h$)	CL [c)] (L/h)	MRT [d)] (h)	Vss [e)] (L)
SSAs	20.48±7.41	48.85±6.63	1.88±0.17	16.31±6.24	43.85±5.37
Ppa	4.29±0.47	2.60±0.11	34.12±9.10	3.64±1.02	180.77±22.76

[a)] $t_{1/2}$: Half-life of the drug; [b)] AUC: Area under the drug concentration; [c)] CL: Clearance rate; d) MRT: Mean residence time; [e)] Vss: Steady-state apparent volume of distribution.

Table S5. Scoring tables of skin reaction.

Erythema

Scores	Observation
0	Normal
1	Slight pink erythema
2	Marked erythema
3	Fiery red erythema
4	Violaceous red erythema and forming crust

Edema

Scores	Observation
0	Normal
1	Slight edema (barely visible)
2	Moderate edema（obvious protrusion）
3	Severe edema (~1mm protrusion with a clear margin)
4	Serious edema (>1mm protrusion with extension)

3 References

[1] Y. H. Feng, X. P. Zhang, Z. Q. Zhao, X. D. Guo, *Mol. Pharm.* **2020**, *17*, 1778.
[2] N. Gong, X. Ma, X. Ye, Q. Zhou, X. Chen, X. Tan, S. Yao, S. Huo, T. Zhang, S. Chen, X. Teng, X. Hu, J. Yu, Y. Gan, H. Jiang, J. Li, X.-J. Liang, *Nat. Nanotechnol.* **2019**, *14*, 379.
[3] Y. Zhang, L. He, J. Wu, K. Wang, J. Wang, W. Dai, A. Yuan, J. Wu, Y. Hu, *Biomaterials* **2016**, *107*, 23.
[4] W. Chung, H. H. Eum, H.-O. Lee, K.-M. Lee, H.-B. Lee, K.-T. Kim, H. S. Ryu, S. Kim, J. E. Lee, Y. H. Park, Z. Kan, W. Han, W.-Y. Park, *Nat. Commun.* **2017**, *8*, 15081.
